# Restoring the Function of Thalamocortical Circuit Through Correcting Thalamic Kv3.2 Channelopathy Normalizes Fear Extinction Impairments in a PTSD Mouse Model

**DOI:** 10.1002/advs.202305939

**Published:** 2023-12-16

**Authors:** Haoxiang Xiao, Kaiwen Xi, Kaifang Wang, Yongsheng Zhou, Baowen Dong, Jinyi Xie, Yuqiao Xie, Haifeng Zhang, Guaiguai Ma, Wenting Wang, Dayun Feng, Baolin Guo, Shengxi Wu

**Affiliations:** ^1^ Department of Neurobiology School of Basic Medicine Fourth Military Medical University Xi'an 710032 China; ^2^ Department of Anesthesiology Tangdu Hospital Fourth Military Medical University Xi'an 710032 China; ^3^ Eastern Theater Air Force Hospital of PLA Nanjing 210000 China; ^4^ Department of Neurosurgery Tangdu Hospital Fourth Military Medical University Xi'an 710032 China

**Keywords:** anterior cingulate cortex, fear extinction, Kv3.2, mediodorsal thalamus, parvalbumin interneuron, post‐traumatic stress disorder

## Abstract

Impaired extinction of fear memory is one of the most common symptoms in post‐traumatic stress disorder (PTSD), with limited therapeutic strategies due to the poor understanding of its underlying neural substrates. In this study, functional screening is performed and identified hyperactivity in the mediodorsal thalamic nucleus (MD) during fear extinction. Furthermore, the encoding patterns of the hyperactivated MD is investigated during persistent fear responses using multiple machine learning algorithms. The anterior cingulate cortex (ACC) is also identified as a functional downstream region of the MD that mediates the extinction of fear memory. The thalamocortical circuit is comprehensively analyzed and found that the MD‐ACC parvalbumin interneurons circuit is preferentially enhanced in PTSD mice, disrupting the local excitatory and inhibitory balance. It is found that decreased phosphorylation of the Kv3.2 channel contributed to the hyperactivated MD, primarily to the malfunctioning thalamocortical circuit. Using a lipid nanoparticle‐based RNA therapy strategy, channelopathy is corrected via a methoxylated siRNA targeting the protein phosphatase 6 catalytic subunit and restored fear memory extinction in PTSD mice. These findings highlight the function of the thalamocortical circuit in PTSD‐related impaired extinction of fear memory and provide therapeutic insights into Kv3.2‐targeted RNA therapy for PTSD.

## Graphical abstract

This study links the overactive mediodorsal thalamic nucleus (MD) in PTSD mice to disrupted fear memory extinction via altered function of the thalamocortical circuit. A lipid nanoparticle RNA therapy targeting thalamic Kv3.2 channel phosphorylation is shown to correct these behavioral abnormalities in PTSD mice, providing a novel therapeutic strategy for PTSD.

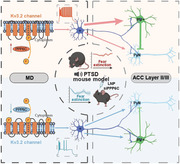

## 1 Introduction

Post‐traumatic stress disorder (PTSD) is a severe psychopathology triggered by extremely aversive events that exacerbate fear responses for extended periods, ranging from months to decades after the traumatic experience.^[^ ^1^, ^2^ ^]^ Such long‐lasting fearful behavioral abnormalities render individuals with PTSD susceptible to psychiatric disorders, such as major depressive disorder, contributing to the high incidence of suicide.^[^ ^3^ ^]^ Thus, the delayed extinction of fear memory in patients with PTSD is believed to be a core instigator of the protracted course of the disease. However, therapeutic strategies to facilitate fear memory extinction remain limited in clinical practice due to a lack of information on neural mechanisms.

Pioneering studies using neuroimaging approaches have identified functional changes in multiple brain areas, including the hippocampus, amygdala, anterior cingulate cortex (ACC), insular cortex, and ventromedial prefrontal cortex, in patients with PTSD.^[^ ^4^, ^5^ ^]^ However, the brain areas and circuits involved in abnormal fear memory extinction are poorly understood. Clinical studies have revealed no differences in the acquisition phase of memory in patients with PTSD,^[^ ^6^, ^7^ ^]^ suggesting the presence of specific areas beyond the well‐known memory control centers in the brain. Exploring these lesser‐known regions has the potential to shed light on the underlying mechanisms of delayed fear memory extinction in PTSD. Examining the functional connectivity and activity patterns within these areas might provide new insights into the neural circuitry involved in the persistence of fear responses, paving the way for developing novel therapeutic strategies to normalize the extinction of fear memories in individuals with PTSD.

Thalamocortical circuit has long been implicated in sensory transmission; however, its broader involvement in affective and cognitive functions is now gaining increased recognition.^[^ ^8^ ^]^ Several studies have highlighted the role of thalamocortical circuit malfunction in various psychiatric disorders, causing behavioral changes such as cognitive and mood deficits.^[^ ^9^, ^10^, ^11^, ^12^ ^]^ Despite emerging neuroimaging evidence indicating vital changes in thalamocortical circuits among patients with PTSD, their precise role remains elusive. Our previous work found that hyperactivity in a specific higher‐order thalamic region, the posterior medial thalamus, contributes to excessive defensive behavior triggered by environmental cues in a mouse model of PTSD.^[^ ^13^ ^]^ In addition to the somatosensory higher‐order thalamus, we observed hyperactivation of the mediodorsal thalamic nucleus (MD), which is implicated in the control of memory processes.^[^ ^14^ ^]^ Moreover, the MD exerts dense innervation on the limbic cortex, including the ACC, which has been extensively investigated as a hub for innate and learned fear.^[^ ^15^ ^]^ Whether this limbic thalamocortical circuitry contributes to fear memory extinction defects in PTSD requires further investigation.

In this study, we examined neuronal activity in the MD and its causality with impaired fear memory extinction employing a well‐established mouse model of PTSD.^[^ ^16^ ^]^ Using a combination of approaches, including optogenetics, chemogenetics, and electrophysiology, we found that hyperactivity in the MD preferentially overactivated downstream parvalbumin‐expressing (PV^+^) interneurons, impairing the local excitatory and inhibitory (E/I) balance in the ACC microcircuitry and consequently leading to delayed fear memory extinction. Mechanistically, we identified abnormal dephosphorylation of the voltage‐gated potassium channel Kv3.2 as a contributing factor to neuronal hyperactivity in the MD. Using a lipid nanoparticle (LNP)‐based RNA therapy strategy, we corrected thalamic channelopathy using a methoxylated siRNA targeting the protein phosphatase 6 catalytic subunit (PPP6C) and rescued fear memory extinction‐related dysfunctional thalamocortical circuits and behavioral abnormalities.

## 2 Results

### 2.1 Hyperactivated Neuronal Activity of the MD in a PTSD Mouse Model

**Figure 1:**
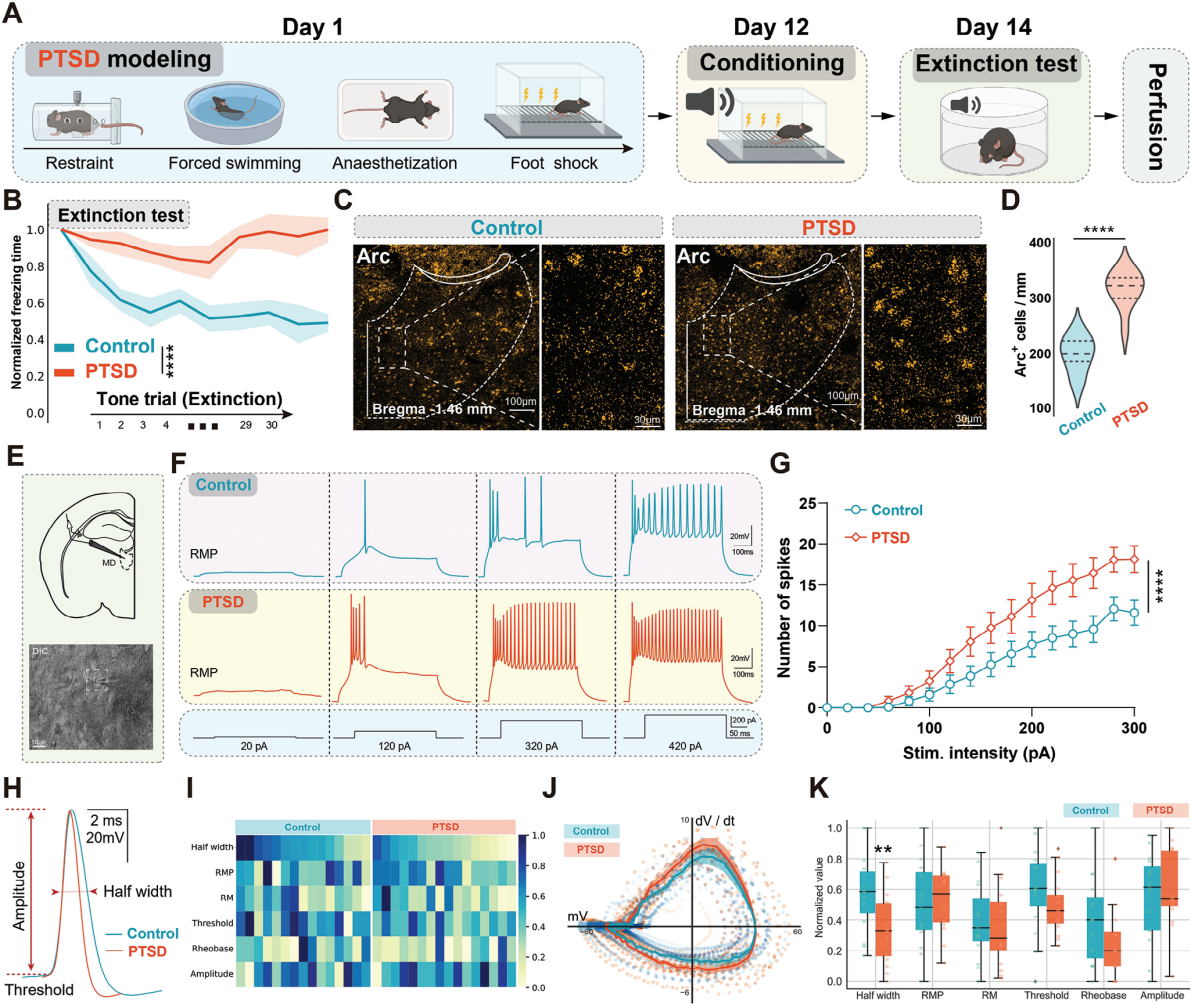
MD neurons were hyperactivated in a mouse model of PTSD. A) Timeline of the experimental procedure. B) Normalized freezing time of mice with PTSD and control mice during the extinction test (*n* = 8 per group, error bar: mean with SEM, ****P < 0.0001 by Friedman's M test with Nimenyi post hoc test). C) Representative image showing Arc expression in MD neurons of control mice and mice with PTSD after extinction stimulus at Bregma, −1.46 mm. Scale bars, 200 µm (left) and 50 µm (right). D) Average Arc+ cell responses to extinction between control mice and mice with PTSD (*n* = 9 slices from three mice per group, error bar: mean with SEM, ****P < 0.0001 by two‐tailed unpaired Student's t‐test). E) Schematic of whole‐cell patch in MD. F) Firing of MD neurons in control mice and mice with PTSD after stimulation with different current intensities under current clamp. G) Average number of spikes of MD neurons between control mice and mice with PTSD under current clamp (*n* = 15–16 neurons from eight mice per group, error bar: mean with SEM, ****P < 0.0001 by two‐way ANOVA with Tukey's post hoc test). H) Representative image showing the half‐width of MD neurons in control mice and mice with PTSD. I) Phase‐plane plot of the first AP at rheobase for all individual neurons in the control and PTSD groups. J) Heatmap of six electrophysiological parameters. K) Average active and passive properties of MD neurons between control mice and mice with PTSD (*n* = 15–16 neurons from eight mice per group, error bar: mean with SEM, **P =  0.0095 by two‐tailed unpaired Student's t‐test). Statistical details are shown in Table S1 (Supporting Information).

We initially established a mouse model of PTSD with single prolonged stress and electric foot shock (SPS&S), which has been well characterized and can effectively replicate the clinical symptoms observed in patients with PTSD^[^ ^16^ ^]^ (**Figure** **1**A). The fear extinction ability of mice with PTSD was assessed by training them to associate a conditioned stimulus (CS), represented by a tone, with an unconditioned stimulus (US), represented by a foot shock. After a 48 h interval, the mice were exposed to CS without US for 30 trials (Figure 1A). Control mice (SPS&S sham) exhibited a gradual decrease in freezing time, suggesting successful fear memory extinction. By contrast, mice with PTSD consistently displayed a high freezing time, indicating impaired fear extinction (Figure 1B).

To investigate changes in activity within thalamic regions, particularly the higher‐order thalamus, which is relevant to cognitive function, we employed the Fos‐TRAP strategy to visualize c‐Fos expression. Tamoxifen was administered 23 h before the extinction test to induce tdTomato expression in Fos‐CreER^T2^;Ai9 mice. We observed increased c‐Fos expression in the MD, paraventricular thalamus, and posterior thalamus among the higher‐order thalamic regions (Figure S1, Supporting Information). We also used another immediate early gene, Arc, to validate our findings and observed an increased number of Arc+ neurons in the MD of mice with PTSD (Figure 1C,D). Additionally, we performed whole‐cell patch‐clamp recordings of MD neurons and found that the firing spike numbers of MD neurons in mice with PTSD were significantly higher than those in wild‐type (WT) mice (Figure 1E–G). Regarding the intrinsic electrophysiological properties, the half‐width of the action potential was significantly lower in the MD neurons of mice with PTSD than in WT mice (Figure 1H–K). We also recorded spontaneous excitatory postsynaptic currents (sEPSCs) in MD neurons and did not observe significant differences in sEPSC frequency or amplitude between control and PTSD groups (Figure S2, Supporting Information). Moreover, we assessed the excitability changes in the MD region using two other well‐established mouse models of PTSD to better confirm the reliability of our findings in SPS&S mouse model.^[^ ^17^, ^18^ ^]^ Through slice recordings on these two PTSD mouse models, we observed higher frequency of action potentials and reduced half‐width, which is very similar to our findings in SPS&S mouse model (Figure S3, Supporting Information).

### 2.2 Hyperactivated MD Encoded Impaired Fear Extinction

**Figure 2:**
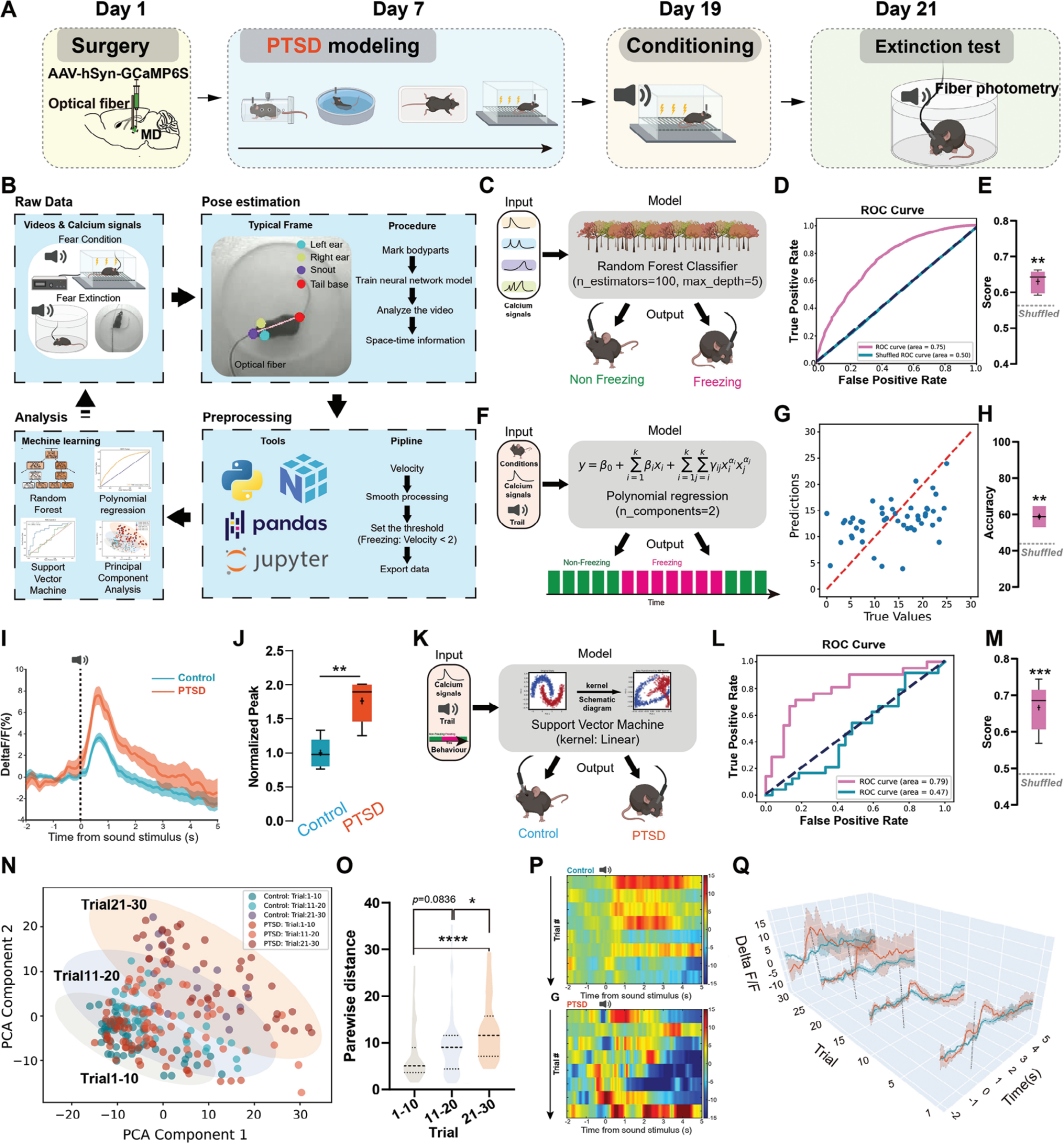
Neuronal activity in the MD correlated with the freezing levels in mice. A) Timeline of the experimental procedure. B) Data analysis flowchart. C) Random forest model schematic diagram. D) Receiver operating characteristic curve. E) Statistical superiority of the predictive performance of the random forest model compared with the control model (*n* = 5 mice per group, error bar: mean with SEM, **P =  0.0031 by two‐tailed unpaired Student's t‐test). F) Schematic diagram of the polynomial regression model. G) Scatter plot of predicted values versus true values. H) Statistical superiority of the predictive performance of the polynomial regression model compared with the control model (*n* = 5 mice per group, error bar: mean with SEM, **P =  0.0081 by two‐tailed unpaired Student's t‐test). I) Temporal progression of MD neuron calcium signals. J) Comparison of bar graphs showing peak calcium signals in MD neurons (*n* = 5 mice per group, error bar: mean with SEM, **P =  0.0011 by two‐tailed unpaired Student's t‐test). K) Illustrative representation of the support vector machine model. L) Receiver operating characteristic curve analysis. M) Predictive performance comparison between models (*n* = 5 mice per group, error bar: mean with SEM, ***P =  0.0007 by two‐tailed unpaired Student's t‐test). N) Plot interpretation of principal component analysis. O) Distance‐based statistical analysis plotting (*n* = 40–50 trials from five mice per group, error bar: mean with SEM, ****P < 0.0001, *P  = 0.0419 by Kruskal–Wallis test with Dunn's post hoc test). P) Heatmap visualization of the MD neuron activity over time. Q) Waterfall chart depicting changes in the MD neuron activity in trials. Statistical details are shown in Table S1 (Supporting Information).

To decode the functional subsequence of the overactivated MD, we performed fiber photometry to record MD calcium signals in vivo. We unilaterally injected an adeno‐associated virus (AAV) encoding a genetically encoded calcium indicator, GCaMP6s, driven by the human synapsin promoter (hSyn), into the MD of PTSD and WT mice. Subsequently, we implanted an optic fiber in the MD and captured behavioral videos to analyze the correlation between MD activity and fear responses (**Figure** **2**A; Figure S5, Supporting Information). The influence of optic fibers on video analysis was minimized by meticulously analyzing the video data using marker‐less pose estimation to predict critical features of mouse movements. In addition to behavioral and calcium activity information, we employed multiple machine learning algorithms, such as random forest models, polynomial regression models, support vector machine models, and principal component analysis, to decode the relationship between MD activity and fear behaviors (Figure 2B; Table S2, Supporting Information).

We initially investigated whether MD activity encodes fear behavior. Using a random forest model trained on over 204255 data points (Figure 2C), we found that MD calcium signals effectively decoded fear behavior in mice on a frame‐by‐frame basis (Figure 2D). Our results highlighted the superior prediction performance of the random forest model compared with the control model (Figure 2E), suggesting that MD played a crucial role in defining fear behavior. However, whether dynamic fluctuations in MD activity can effectively predict the timing of fear behavior remains unclear. To address this question further, we used a polynomial regression model to predict the duration of freezing behavior in each trial. This model incorporated variables such as the conditions of the mice, MD calcium signals, and the number of trials (Figure 2F). By generating a scatter plot of predicted versus actual values, we observed a linear distribution of data points (Figure 2G). Compared with the control model, the analysis suggested that the predictive accuracy of the polynomial regression model was notably superior (Figure 2H). These findings indicate that MD encodes fear behavior and significantly contributes to the severity of fear behavior.

We analyzed the calcium signal peak following the tone in both mice with PTSD and control mice and found that the peak amplitude was significantly higher in mice with PTSD (Figure 2I,J). We then utilized a support vector machine model to decode mouse conditions based on their MD neuronal calcium signals and corresponding behaviors (Figure 2K). The decoder showed high accuracy in predicting mouse conditions, especially when compared with the control (Figure 2L,M). To better understand the difference between mice with PTSD and control mice, we utilized principal component analysis to simplify and visually represent multifaceted datasets, including MD neuron activity, mouse fear behavior patterns, and general mouse conditions (Figure 2N). By calculating the pairwise distance between the points from the two groups, we found an increasing widening distance as the number of extinction trials increased, particularly in the last 10 trials (Figure 2O). This difference was also evident when the MD calcium responses for each trial were plotted (Figure 2P,Q). We also recorded the neuronal activity of MD while exposed to fear‐inducing stimuli and found the PTSD mouse showed an overactivated response compared to control group (Figure S4, Supporting Information). These results indicate that abnormal MD activity in mice with PTSD encodes persistent fear behaviors during the extinction test.

### 2.3 Optogenetic Inhibition of MD Promoted Fear Extinction

**Figure 3:**
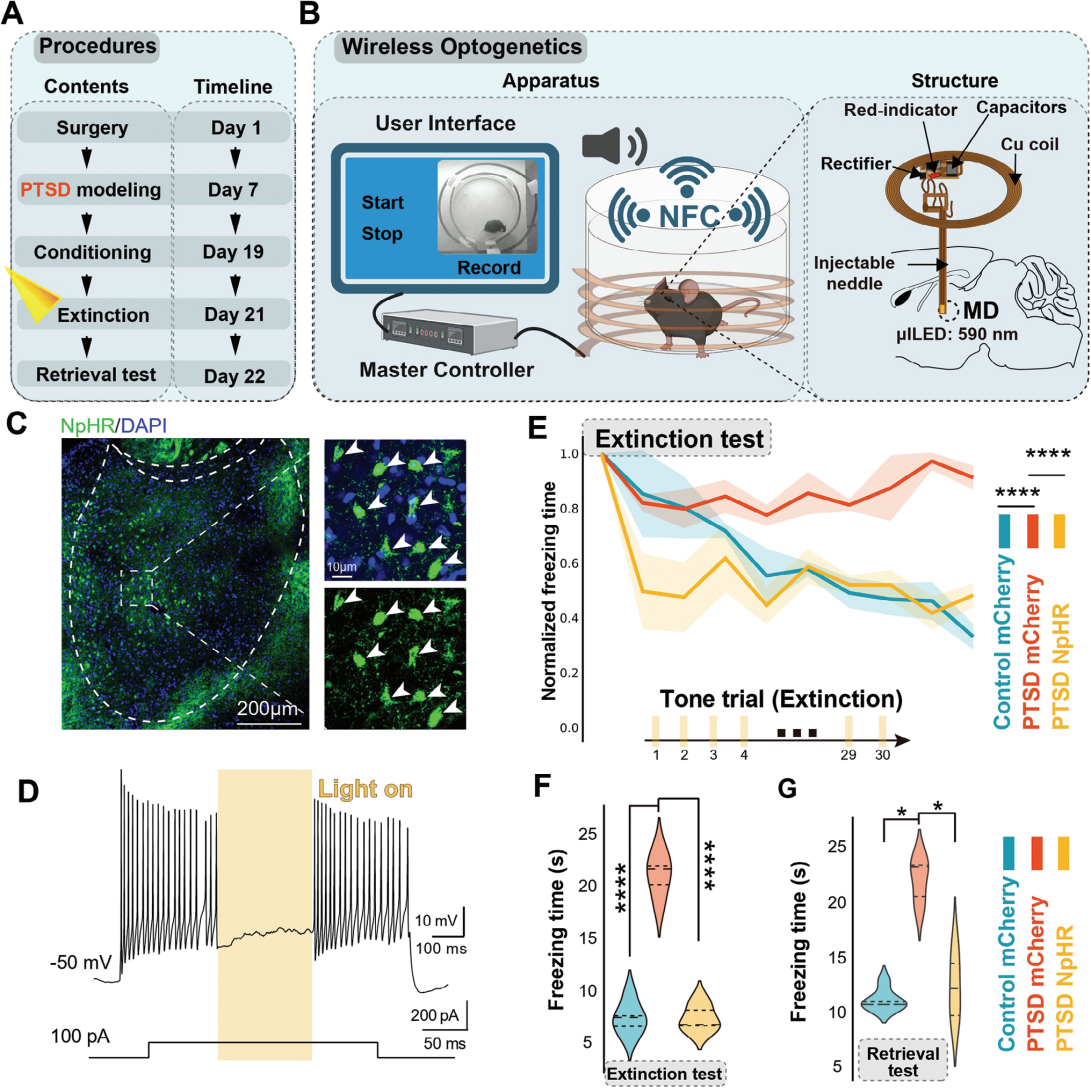
Wireless optogenetic inhibition of MD neurons induced decreased freezing levels during the extinction test in mice with PTSD. A) Timeline of the experimental procedure. B) Illustration of wireless optogenetics. C) Representative images showing the distributions of NpHR‐labeled neurons in the MD. D) Whole‐cell voltage clamp showing light‐mediated silencing of MD neurons in an acute slice preparation. E) Normalized freezing time responses to fear extinction between control mice and mice with PTSD (*n* = 5 mice per group, error bar: mean with SEM, ****P < 0.0001 by Friedman's M test with Nimenyi post hoc test). F) Wireless optogenetic inhibition of MD activity‐induced decreased freezing levels in mice with PTSD during the extinction test (*n* = 5 mice per group, error bar: mean with SEM, ****P < 0.0001 by one‐way ANOVA with Tukey's post hoc test). G) Average freezing time responses to the retrieval test between control mice and mice with PTSD (n = 5 mice per group, error bar: mean with SEM, *P_Control mCherry versus PTSD mCherry_ = 0.0232, *P_PTSD mCherry versus PTSD NpHR_ = 0.0232 by Kruskal–Wallis test with Dunn's post hoc test). Statistical details are shown in Table S1 (Supporting Information).

Given the causal role of hyperactivated MD in persistent fear behaviors during extinction, we determined whether direct optogenetic inhibition of MD could promote fear extinction in mice with PTSD. We delivered eNpHR 3.0, a light‐activated chloride‐pumping halorhodopsin, to MD neurons (**Figure** **3**A,B) by using a wireless optogenetic device, as previously described.^[^ ^19^ ^]^ This approach aimed to minimize the influence of optic fibers and facilitate behavioral analysis. The battery‐free wireless device included microscale inorganic light emitting diodes and radiofrequency strategies for wireless power delivery, enabling fully implantable, tether‐free optogenetic studies (Figure 3B). We injected the AAV2/9‐hSyn‐eNpHR3.0‐EYFP or AAV2/9‐hSyn‐mCherry control virus into the MD and implanted the penetrating probe into the same site (Figure 3C; Figure S6, Supporting Information). To verify the effectiveness of eNpHR, we used the whole‐cell patch‐clamp technique to record the firing of MD neurons after stimulation with yellow light (590 nm) under current clamp. Firing of MD neurons was immediately suppressed during yellow laser stimulation (Figure 3D). Our results showed that optogenetic inhibition of MD neurons significantly reduced freezing time in mice with PTSD during fear extinction (Figure 3E,F) and decreased fearful responses in the retravel test (Figure 3G). These observations suggest that inhibiting MD neuronal activity could promote fear extinction in mice with PTSD.

### 2.4 Hyperactivated MD Functionally and Structurally Reshaped the Local Microcircuitry in the ACC

**Figure 4:**
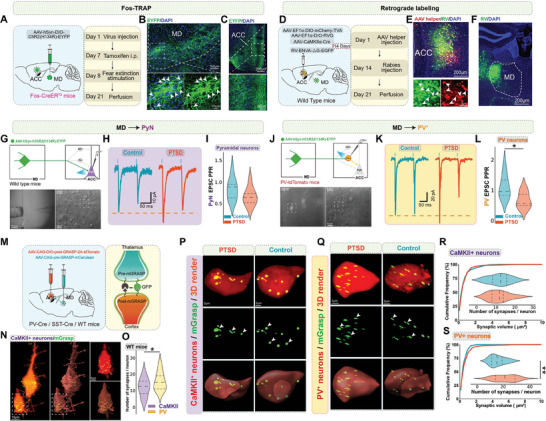
MD preferentially projected to PV^+^ neurons in the ACC during fear extinction in mice with PTSD. A) Timeline and illustration of the Fos‐TRAP procedure. B,C) Coronal sections of MD and ACC with ChR2‐EYFP; scale bar, left: 100 µm (top), 10 µm (bottom); right: 200 µm (top), 50 µm (bottom). D) Timeline and illustration of the RV injection. E,F) Coronal sections of ACC and MD with RV; scale bar, left: 200 µm (top), 50 µm (bottom); medium: 50 µm; right: 200 µm. G,J) Schematics of the paired‐pulse ratio procedure. Representative paired‐pulse traces H) and quantitative summary I) of CaMKII^+^ neurons showing no significant differences (*n* = 9–10 neurons from three mice per group, error bar: mean with SEM, ns = 0.1368 by two‐tailed unpaired Student's t‐test). Representative paired‐pulse traces K) and quantitative summary L) of PV^+^ interneurons showing reduced PPR in mice with PTSD (*n* = 7–8 neurons from three mice per group, error bar: mean with SEM, *P = 0.0233 by two‐tailed unpaired Student's t‐test). M) Schematic of synaptic labeling mechanism using PV‐Cre driver lines, SST‐Cre driver lines, or the CaMKII‐Cre virus to label inhibitory or excitatory synapses. N) High‐magnification confocal image of a post‐mGRASP‐labeled CaMKII^+^ neuron (red) in the ACC, merged with synaptic labeling using mGRASP (green). Arrowheads indicate the putative synapses. Scale bars, 10 µm (left and middle). O) Quantification of thalamocortical synaptic frequency in WT mice, showing that PV^+^ neurons in the ACC received a significantly higher frequency of thalamocortical synapses than CaMKII^+^ neurons (*n* = 28–30 neurons from three mice per group, error bar: mean with SEM, *P = 0.0484 by Mann–Whitney U test). P) High‐magnification confocal (top) and 3D images (middle and bottom) of a post‐mGRASP‐labeled CaMKII^+^ neuron (red) in the ACC, merged with synaptic labeling using mGRASP (green). Arrowheads indicate the putative synapses. Scale bars, 3 µm. Q) High‐magnification confocal (top) and 3D images (middle and bottom) of a post‐mGRASP‐labeled PV^+^ neuron (red) in the ACC, merged with synaptic labeling using mGRASP (green). Arrowheads indicate the putative synapses. Scale bars, 3 µm. R) Quantification of thalamocortical synaptic frequency in WT mice showing that CaMKII^+^ neurons in the ACC had a significant difference in the frequency of thalamocortical synapses between control mice and mice with PTSD (*n* = 30–33 neurons from four mice per group, error bar: mean with SEM, ns = 0.8070 by two‐tailed unpaired Student's t‐test, ns > 0.999 by Kolmogorov‐Smirnov D test). The cumulative frequency distribution of synapses by volume in CaMKII^+^ neurons in the ACC showed no significant difference between the thalamocortical synapses in control mice and mice with PTSD. S) Quantification of thalamocortical synaptic frequency in WT mice, showing that PV^+^ neurons in the ACC received a significantly higher frequency of thalamocortical synapses in mice with PTSD than in control mice (*n* = 28 neurons from three mice per group, error bar: mean with SEM, **P = 0.0038 by Mann–Whitney U test). The cumulative frequency distribution of synapses by volume in PV^+^ neurons in the ACC showed no significant differences between the thalamocortical synapses in control mice and mice with PTSD. Statistical details are presented in Table S1 (Supporting Information).

To determine the functional downstream effects of the MD during fear extinction, we used the Fos‐TRAP strategy combined with anterograde tracing. This approach enables precise Cre expression on neurons activated by specific stimuli in the time window of tamoxifen injection. We initially injected AAV2/9‐hSyn‐DIO‐ChR2‐EYFP into the MD of Fos‐CreER^T2^ mice. Tamoxifen (100 mg kg^−1^) was intraperitoneally injected to the mice 23 h before fear extinction (**Figure** **4**A). The ACC exhibited dense axons expressing EYFP (Figure 4B,C). We injected rabies virus (RV) into the ACC and observed fluorescently labeled neurons in the MD, verifying the anatomical connection between the MD and ACC (Figure 4D–F).

Next, we examined the influence of hyperactivated MD on synaptic strength in the thalamocortical circuit. Using optogenetic stimulation, we recorded pyramidal neurons, PV^+^ interneurons, and somatostatin‐expressing (SST^+^) interneurons to test the input strength from the MD (Figure 4G–L). Interestingly, a detectable difference in the paired‐pulse ratio (PPR) was observed only in PV^+^ interneurons between the control and PTSD groups, not in the pyramidal cells and SST^+^ interneurons (Figure 4I,L; Figure S7A–C, Supporting Information). Previous studies showed that strong synaptic activation can induce the formation of new synapses and activity‐dependent plasticity.^[^ ^20^, ^21^ ^]^ We tested whether enhanced MD synaptic inputs reshaped local synaptic connectivity in the ACC by employing mammalian GFP reconstitution across synaptic partners (mGRASP). This technique is based on two functionally complementary non‐fluorescent GFP fragments expressed in pre‐ and post‐synaptic neurons, specifically labeling the synapses between the two neuronal populations for more accurate and specific visualization (Figure 4M).^[^ ^22^ ^]^ In control mice, we observed a greater number of synapses formed by MD axons and PV^+^ neurons in the ACC compared with pyramidal neurons, implying a potentially stronger connection between MD and ACC PV^+^ neurons (Figure 4N,O). In mice with PTSD, an increased number of thalamocortical synapses were observed in PV^+^ interneurons (Figure 4Q,S). However, the synapses in pyramidal and SST^+^ neurons remained comparable with those in the control mice (Figure 4P–R; Figure S7D,E, Supporting Information). Notably, the difference in dynamic changes in the number of synapses between the two groups was significant, but not in the size of the synapses.

**Figure 5:**
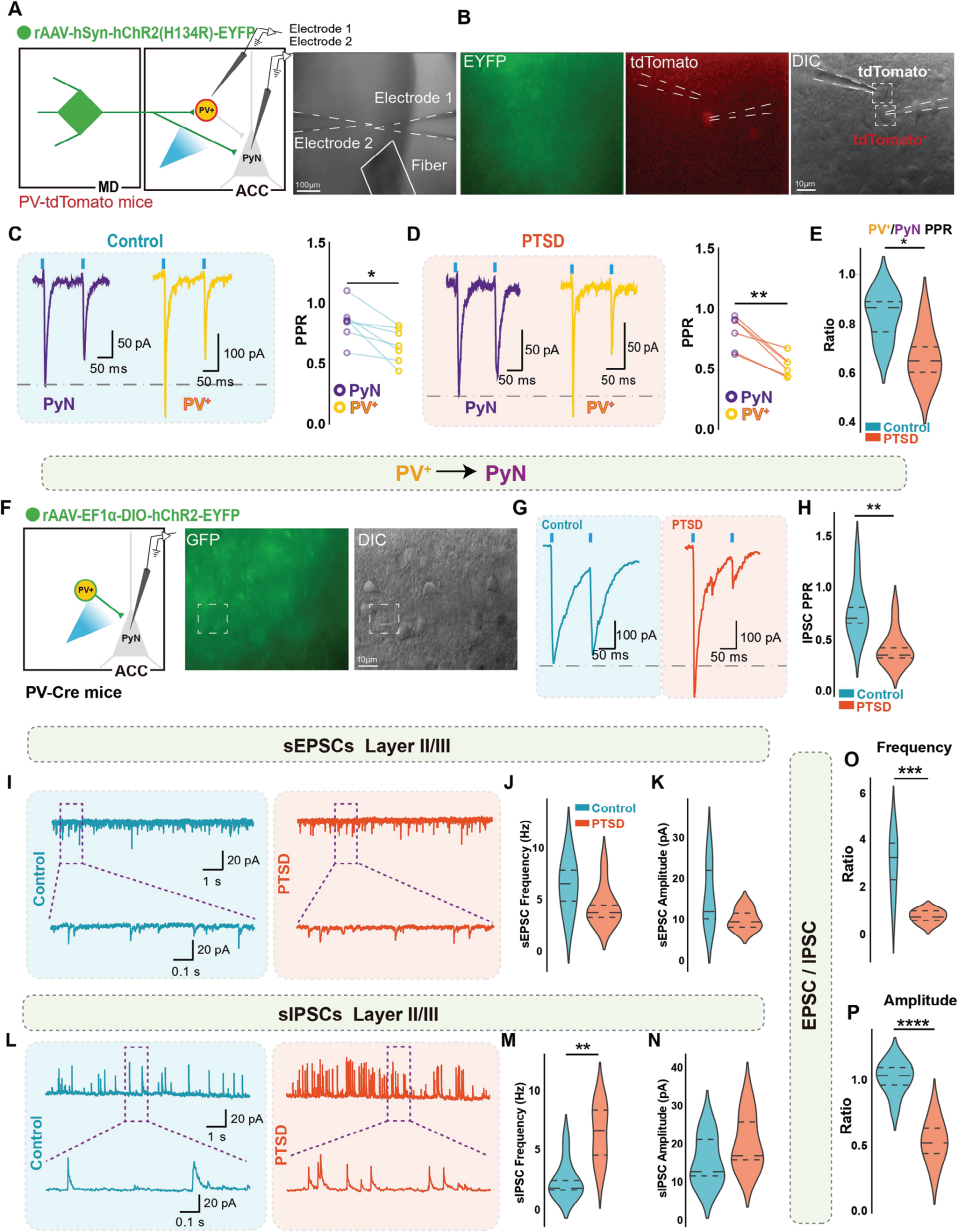
Excessive activation of PV^+^ neurons in ACC layer II/III increased sIPSC frequency in pyramidal neurons. A,B) Schematic of whole‐cell double patch in the ACC. Scale bar, 100 µm A), 10 µm B). C) Representative paired‐pulse traces (left) and quantitative summary (right) of pyramidal and PV^+^ neurons in the control group showing reduced PPR in PV^+^ neurons (*n* = 6 neurons from four mice per group, error bar: mean with SEM, *P = 0.0178 by two‐tailed paired Student's t‐test). D) Representative paired‐pulse traces (left) and quantitative summary (right) of pyramidal and PV+ neurons in the PTSD group showing reduced PPR in PV^+^ neurons compared with the control group (*n* = 6 neurons from four mice per group, error bar: mean with SEM, **P = 0.0026 by two‐tailed paired Student's t‐test). E) Quantitative summary of PV/PyN PPR showing a reduced ratio in the PTSD group (*n* = 6 neurons from four mice per group, error bar: mean with SEM, *P = 0.0169 by two‐tailed unpaired Student's t‐test). F) Schematic of the whole‐cell patch in the ACC. Scale bar, 10 µm. Representative paired‐pulse traces G) and quantitative summary H) of pyramidal neurons in the ACC showing reduced PPR in the PTSD group (*n* = 8–9 neurons from three mice per group, error bar: mean with SEM, **P = 0.0037 by Mann–Whitney U test). I–K) Representative traces I), quantification of sEPSC frequency J), and amplitude K) showing no significant differences in frequency and amplitude between the control and PTSD groups (*n* = 8 neurons from five to six mice per group, error bar: mean with SEM, ns_J_ = 0.1304, ns_K_ = 0.0830 by Mann–Whitney U test). L–N) Representative traces L) and quantification of sIPSC frequency M) and amplitude N) showing a significant increase in sIPSC frequency but not in amplitude (*n* = 8 neurons from five to six mice per group, error bar: mean with SEM, **P = 0.0030 by Mann–Whitney U test). O) Quantification of E/I frequency ratio and P) E/I amplitude ratio showing a significantly decreased E/I ratio in frequency and amplitude in mice with PTSD (*n* = 8 neurons from five to six mice per group, error bar: mean with SEM, ***Po *=* 0.0002, ****P_P_ < 0.0001 by two‐tailed unpaired Student's t‐test). Statistical details are shown in Table S1 (Supporting Information).

Next, we investigated whether the enhanced connectivity between the MD and ACC influenced the local microcircuit in the ACC owing to the preferential alterations observed in different cell types. First, we performed dual whole‐cell recordings in pyramidal cells and PV^+^ interneurons in a PV‐tdTomato transgenic mouse to simultaneously test the synchronization of the two types of neurons in response to MD synaptic information (**Figure** **5**A,B). By measuring the PPR, we found that PV^+^ cells presented a lower PPR than pyramidal neurons in control mice and mice with PTSD, consistent with our previous observations of synapses (Figure 5C,D). We calculated the percentage of PPR collected from pairs of PV^+^ interneurons and pyramidal cells. The cell pairs from mice with PTSD showed a lower PPR than those from control mice, indicating a relatively increased thalamic information to PV^+^ interneurons in the ACC in mice with PTSD (Figure 5E). Subsequently, we directly tested the synaptic inputs to pyramidal neurons in the ACC derived from PV^+^ interneurons and found a decreased PPR in mice with PTSD (Figure 5F–H). These results indicate that hyperactivated MD neurons augmented local inhibition in the ACC by preferentially increasing synaptic inputs to PV^+^ interneurons.

We investigated whether imbalanced E/I synaptic information could be layer‐specific in the ACC. Using anterograde tracing, we observed more MD axons in layer II/III than in the other layers (Figure S8, Supporting Information). Functionally, we recorded the sEPSCs and spontaneous inhibitory postsynaptic currents (sIPSCs) in the same cell from layers II/III and V/VI (Figure 5I–N). We only observed an increased frequency of sIPSCs in pyramidal cells from layer II/III in mice with PTSD, resulting in a decreased frequency and amplitude ratio of sEPSCs/sIPSCs (Figure 5M,O,P). No detectable changes were detected in layer V/VI (Figure S9, Supporting Information). Furthermore, the intrinsic properties of pyramidal neurons in layers II/III and V/VI showed no significant changes in mice with PTSD (Figure S10, Supporting Information). The intrinsic properties of SST^+^ and PV^+^ interneurons in layer II/III showed no significant changes in mice with PTSD (Figures S11 and S12, Supporting Information). Taken together, these results suggest that increased thalamocortical synaptic inputs reshaped the local microcircuitry in the ACC and enhanced local inhibition in mice with PTSD.

### 2.5 Critical Role of PV^+^ Interneurons in MD‐ACC Circuit in Mediating Abnormal Fear Extinction

**Figure 6:**
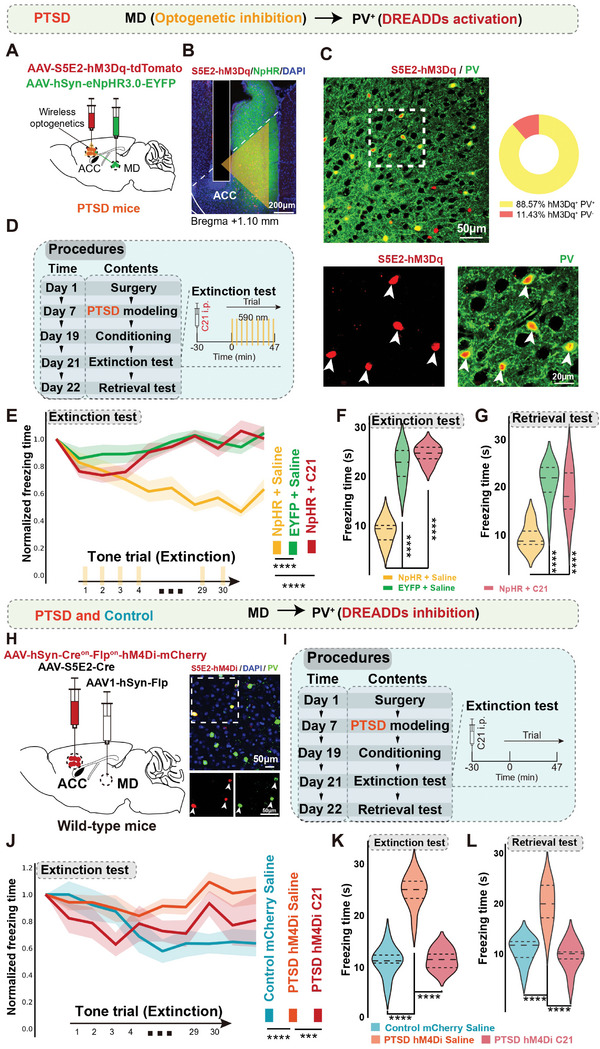
Excessive excitation of PV^+^ neurons in the ACC projecting from the MD was sufficient and necessary for the higher freezing levels of mice with PTSD. A) Schematic of virus injection. B) Representative images showing the distribution of eNpHR and S5E2‐hM3D(Gq)‐labeled neurons and the fiber placement in the ACC. Scale bar, 200 µm. C) Representative images of co‐labeling for PV (green) and S5E2‐hM3D(Gq) (red) in the ACC. Scale bar, 50 µm (left), 20 µm (middle and right). D) Timeline of the experimental procedure. E) Optogenetic inhibition (589 nm light) of the MD induced decreased freezing levels during the extinction test in the PTSD NpHR group compared with the PTSD EYFP group. Chemogenetic activation of PV^+^ neurons in the ACC reversed freezing levels in the PTSD NpHR group (*n* = 10 mice per group, error bar: mean with SEM, ****P < 0.0001 by two‐way ANOVA with Tukey's post hoc test). Quantification summary of the extinction test F) and retrieval test G) showing that inhibition of MD activity decreased freezing levels, whereas activation of PV^+^ neurons in the ACC reversed these results (*n* = 10 mice per group, error bar: mean with SEM, ****P < 0.0001 by one‐way ANOVA with Tukey's post hoc test). H) Schematic of virus injection. Representative images showing the distribution of S5E2‐hM4D(Gi)‐labeled neurons in the ACC. Scale bar, 50 µm. I) Timeline of the experimental procedure. J) Chemogenetic inhibition of PV^+^ neurons in the ACC projecting from the MD decreased freezing levels of mice with PTSD during the extinction test (*n* = 9–10 mice per groups, error bar: mean with SEM, ****P _Control mCherry Saline versus PTSD hM4Di Saline_ < 0.0001, ***P_PTSD hM4Di Saline versus PTSD hM4Di C21_ < 0.001 by Friedman's M test with Nimenyi post hoc test). Quantification summary of the extinction test K) and retrieval test L) showing that inhibition of PV^+^ neurons in the ACC projecting from the MD decreased freezing levels of mice with PTSD (*n* = 9–10 mice per groups, error bar: mean with SEM, ****P < 0.0001 by one‐way ANOVA with Tukey's post hoc test). Statistical details are shown in Table S1 (Supporting Information).

To further verify the role of PV^+^ interneurons downstream of the MD in regulating abnormal fear extinction related to PTSD, we performed in vivo optogenetic and chemogenetic manipulations. We initially expressed eNpHR 3.0 in MD cells and implanted fibers into the MD for photo‐inhibition. Next, we injected an AAV harboring hM3D(Gq), driven by the PV‐specific enhancer S5E2, into the ACC (**Figure** **6**A,B; Figure S13, Supporting Information). All animals were subjected to PTSD and injected with S5E2‐hM3D(Gq) in the ACC. During immunostaining of brain slices containing the ACC with PV antibodies to verify the specificity of the PV enhancer, 88.57% of neurons were labeled with PV (Figure 6C). For the NpHR+C21 group, we administered yellow light (590 nm) stimulation throughout fear extinction and intraperitoneally injected C21 30 min before the fear extinction (Figure 6D). We also used the EYFP+Saline and NpHR+Saline groups as controls. Animals in the NpHR+Saline group showed normal extinction during the extinction test, whereas animals in the NpHR+C21 group exhibited diminished rescue effects of optogenetic MD inhibition (Figure 6E–G). These results suggest that the activation of PV^+^ interneurons was necessary for MD‐dependent fear extinction regulation.

To further characterize the role of MD‐ACC PV^+^ interneurons in fear extinction impairment related to PTSD, we bilaterally injected a high‐titer AAV2/1‐Flp virus expressing Flp transsynaptically into the MD. Subsequently, using the Cre^on^ Flp^on^ system, we introduced Cre into PV^+^ interneurons in the ACC with another AAV expressing hM4D(Gi), owing to the co‐expression of Cre and Flp (Figure 6H; Figure S14, Supporting Information). C21 or saline was intraperitoneally injected in mice with PTSD and control mice 30 min before the fear extinction test to inhibit ACC PV^+^ neurons with MD innervation (Figure 6I). During the behavioral test, C21 administration significantly promoted fear extinction in hM4D(Gi)‐expressing mice with PTSD compared with saline injection, and the extinction level was comparable with that of control mice (Figure 6J–L). These results suggest a critical role for MD‐ACC PV^+^ interneurons in regulating fear extinction in mice with PTSD.

### 2.6 Decreased Phosphorylation of Kv3.2 in the MD

**Figure 7:**
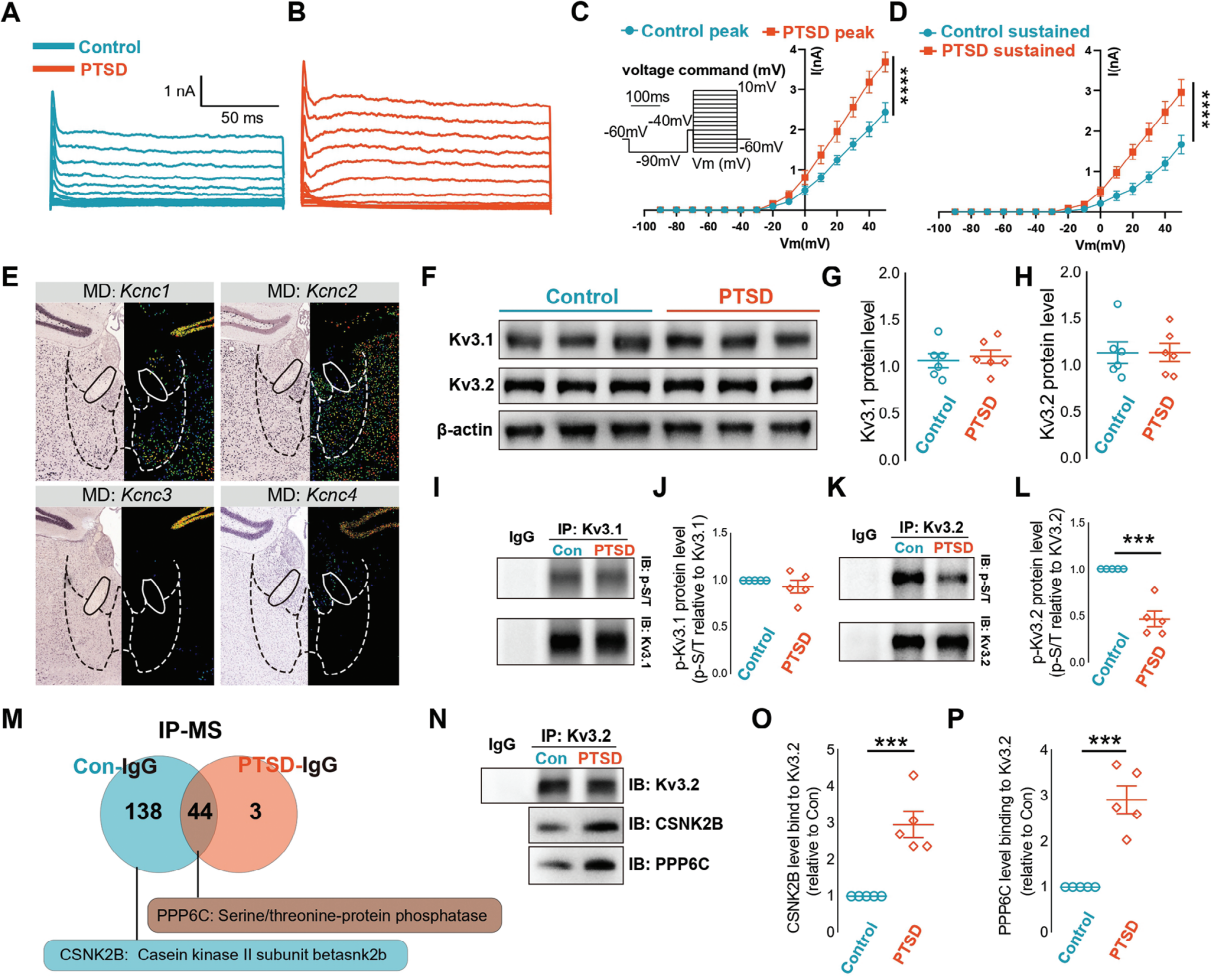
Increased PPP6C expression led to enhanced Kv3.2 channel function. A,B) Representative traces from voltage commands from −90 to +40 mV are shown for the Kv3 channel of MD neurons. C,D) I/V curves of peak currents for MD neurons, from the control mice and mice with PTSD, respectively, showing a higher Kv3 current in mice with PTSD than in control mice (*n* = 8 neurons from four mice per group, error bar: mean with SEM, ****P < 0.0001 by Friedman's M test with Nimenyi post hoc test). E) Kcnc1, Kcnc2, Kcnc3, and Kcnc4 mRNA expression (coronal sections) revealed by in situ hybridization (ISH) (data from Mouse Brain Atlas, Allen Institute of Brain Science; Kcnc1, Exp. 72 108 825; Kcnc2, Exp. 73 512 364; Kcnc3, Exp. 71 670 481; Kcnc4, Exp. 1756). F–H) Representative immunoblots and quantitative summary of Kv3.1 and Kv3.2 levels in MD lysates from control mice and mice with PTSD (*n* = 6 mice per group, error bar: mean with SEM, ns_G_ = 0.6881 and ns_H_ = 0.9858 by two‐tailed unpaired Student's t‐test). I,J) Representative immunoblots of immunoprecipitated p‐S/T and quantitative summary of p‐S/T and Kv3.1 levels (*n* = 5 mice per group, error bar: mean with SEM, ns = 0.3635 by two‐tailed unpaired Student's t‐test). K,L) Representative immunoblots of immunoprecipitated p‐S/T and quantitative summary of p‐S/T and Kv3.2 levels (*n* = 5 mice per group, error bar: mean with SEM, ***P = 0.0002 by two‐tailed unpaired Student's t‐test). M) Schematic of mass spectrometry analysis. N–P) Representative immunoblots and quantitative summary of immunoprecipitated Kv3.2, CSNK2B, and PPP6C in MD lysates derived from control mice and mice with PTSD (*n* = 5 mice per group, error bar: mean with SEM, ***P_O_ = 0.0006, ***P_P_  = 0.0002 by two‐tailed unpaired Student's t‐test). Statistical details are presented in Table S1 (Supporting Information).

Because hyperactivated MD is the main contributor to circuitry alterations in PTSD, we examined the substrates of neuronal hyperactivity. The most evident change in the intrinsic activity of MD cells was a reduced half‐width, which was reported to be mediated by voltage‐gated K+ and Kv3 channels. Kv3 channels play a role in the rapid repolarization of action potentials in neurons.^[^ ^23^ ^]^ Changes in Kv3 channels were detected by recording Kv3 currents in MD neurons of mice with PTSD and WT mice using whole‐cell patch‐clamp techniques. The results indicated higher Kv3 current intensity in mice with PTSD than in WT mice (**Figure** **7**A–D). Among the Kv3 family, Kv3.1 and Kv3.2 were expressed in the MD (Figure 7E), with no observed changes in their protein levels between the PTSD and control groups (Figure 7F–H; Figure S15, Supporting Information). A previous study reported that phosphorylation of the Kv3 channel could influence its currents.^[^ ^24^ ^]^ We analyzed Kv3.1 and Kv3.2 phosphorylation levels and found that the Kv3.2 channel phosphorylation level in MD neurons of mice with PTSD was significantly lower than that in WT mice, but that of the Kv3.1 channel remained unchanged (Figure 7I–L; Figure S15, Supporting Information).

We performed immunoprecipitation‐mass spectrometry analysis to identify potential molecules regulating Kv3.2 phosphorylation. Two related upregulated proteins, casein kinase II beta subunit (CSNK2B) and PPP6C, were identified, and their levels were significantly increased in mice with PTSD (Figure 7M–P; Figure S15, Supporting Information). Protein kinases and phosphatases primarily regulate phosphorylation. Hence, the upregulation of PPP6C likely results in decreased phosphorylation of Kv3.2.

### 2.7 Conditional Knockdown PPP6C in the MD Effectively Facilitated Fear Extinction

**Figure 8:**
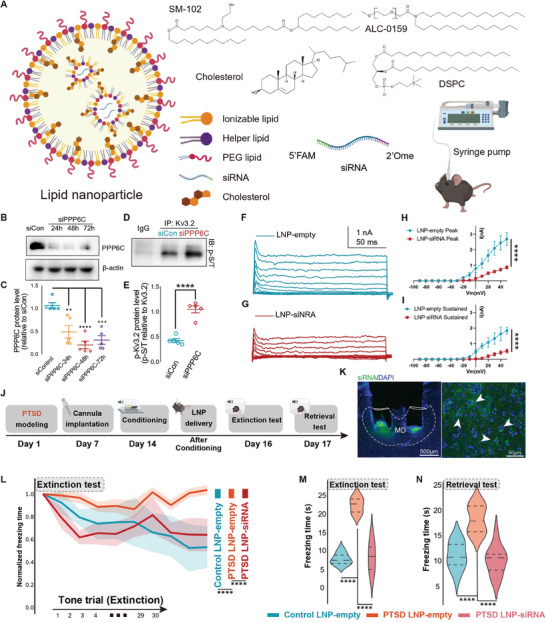
LNP‐siRNA treatment reversed high freezing levels in a mouse model of PTSD. A) Illustration of LNP‐siRNA envelope. B) Representative immunoblots and C) quantitative summary of Kv3.2 protein levels in MD lysates from the siControl and siPPP6C groups (*n* = 5 mice per group, error bar: mean with SEM, **P_siControl versus siPPP6c‐24 h_ = 0.0021, ****P_siControl versus siPPP6c‐48 h_ < 0.0001, ***P_siControl versus siPPP6c‐72 h_ = 0.0002 by one‐way ANOVA with Tukey's post hoc test). D) Representative immunoblots of immunoprecipitated p‐S/T and E) quantitative summary of p‐S/T levels from the siControl and siPPP6C groups (*n* = 5 mice per group, error bar: mean with SEM, ****P < 0.0001 by two‐tailed unpaired Student's t‐test). F,G) Representative traces from voltage commands from −90 to +40 mV are shown for the Kv3 channel of MD neurons between the control and siRNA groups. H,I) I/V curves of peak currents for MD neurons from the control and siRNA mice (*n* = 6–7 neurons from 3 mice per group, error bar: mean with SEM, ****P < 0.0001 by Friedman's M test with Nimenyi post hoc test). J) Timeline and illustration of the LNP procedure. K) Representative images showing the distributions of the cannula placement site and LNP‐siRNA labeled neurons in MD. Scale bar, 500 µm (left), 50 µm (right). L) LNP‐siRNA knockdown of PPP6C decreased freezing levels of PTSD mice during the extinction test (*n* = 10 mice per group, error bar: mean with SEM, ****P < 0.0001 by Friedman's M test with Nimenyi post hoc test). Quantification summary of extinction M) and retrieval N) tests showing that knockdown of PPP6C in MD neurons decreased freezing levels in mice with PTSD (*n* = 10 mice per group, error bar: mean with SEM, ****P < 0.0001 by one‐way ANOVA with Tukey's post hoc test). Statistical details are shown in Table S1 (Supporting Information).

Next, we conditionally knocked down PPP6C expression in the MD and tested whether it could facilitate fear extinction in mice with PTSD. We constructed four PPP6C siRNAs and selected the siRNA with the highest efficiency (Figure S16, Supporting Information). To improve the in vivo transfection efficiency of PPP6C siRNA, we modified it with 2′‐*O*‐methyl. We packaged the siRNA into a non‐viral system of LNPs and bilaterally infused it into the MD using a cannula (**Figure** **8**A). We tested the biophysical characteristics of LNP and assayed the stability of LNP‐siRNA, supporting that the LNP system was capable for in vivo siRNA delivery (Figure S17 and Table S3, Supporting Information). The time window for LNP‐siRNA delivery was optimized by testing the protein level of PPP6C at 24, 48, and 72 h following local infusion (Figure 8B,C; Figure S18, Supporting Information). We selected the 48‐h time window for behavioral perturbation and validated the phosphorylation of Kv3.2 and Kv3 48 h after LNP‐siRNA administration (Figure 8D,E; Figure S18, Supporting Information). The Kv3.2 current intensity was significantly lower in the LNP‐siRNA group than in the LNP‐empty group (Figure 8F–I). siRNA also suppressed neuronal firing by decreasing the half‐width of the action potentials (Figure S19, Supporting Information). In addition, we recorded the PV^+^ neurons with PV‐tdTomato mice, and use optogenetic stimulation to test the connectivity. We detected an increasing PPR in the PTSD LNP‐siRNA group, suggesting that the LNP‐siRNA reduced the thalamic inputs to ACC PV^+^ neurons (Figure S20, Supporting Information). Behaviorally, we observed that LNP‐siRNA administration significantly promoted fear extinction in the PTSD mouse model compared with the empty‐LNP group (Figure 8H–J; Figure S21, Supporting Information). Thus, our results demonstrated that LNP‐siRNA could inhibit PPP6C in MD neurons and rescue the impairment of fear extinction in a PTSD mouse model.

## 3 Discussion

Long‐standing impairment in fear extinction has significantly impacted patients, seriously affecting their daily lives. Numerous functional imaging studies have revealed decreased volumes in multiple brain areas in patients with PTSD. However, identifying the specific region that plays the dominant role proves to be challenging. Clinical studies have demonstrated that changes in thalamic nuclei volume in adults after trauma are associated with trauma reexperiencing.^[^ ^25^, ^26^ ^]^ Preclinical evidence based on brain magnetic resonance imaging has shown the presence of atrophic thalamic nuclei after stress in a mouse model.^[^ ^27^ ^]^ Compelling evidence suggests that the thalamus plays other critical roles besides sensory information relay.^[^ ^28^, ^29^ ^]^ For example, the thalamic reticular nucleus feedforward inhibition of the lateral hypothalamus controls arousal and consciousness.^[^ ^30^ ^]^ Additionally, decreased MD activity disrupts prefrontal‐dependent cognitive behavior in individuals with schizophrenia.^[^ ^31^ ^]^

Furthermore, previous studies reported thalamic activation in response to fearful stimulation,^[^ ^32^ ^]^ which is consistent with our findings of excessive activation in the MD during fear extinction in a PTSD mouse model. Increased functional connections between the ACC and thalamus have also been detected in patients with PTSD.^[^ ^33^ ^]^ Other studies reported a depletion of functional connectivity between the ACC and thalamus in patients with PTSD.^[^ ^34^, ^35^ ^]^ Therefore, elucidating the role of the cingulate thalamocortical circuit in fear extinction is important using a PTSD model. A comprehensive understanding of the molecular details of the thalamocortical circuit involved in PTSD holds promise for developing targeted therapeutic interventions. In this study, by unraveling the intricate interactions within the thalamocortical circuitry, we identified a novel target that could potentially restore normal fear memory extinction in a mouse model of PTSD. Our findings expand the understanding of the neural substrates involved in PTSD‐related fear extinction impairment and provide therapeutic insights supported by preclinical evidence.

Fear extinction is a complex process that involves reducing conditioned fear responses to previously acquired fear stimuli.^[^ ^36^ ^]^ The underlying mechanisms involve various brain regions that exhibit plasticity associated with extinction, together with the intricate cellular and molecular processes in the extinction process. The thalamocortical circuit is implicated in the regulation of associative memory. A previous study confirmed that high‐frequency burst firing in MD neurons prevents fear extinction.^[^ ^37^ ^]^ We provide evidence establishing a causal link between hyperactivated MD and impaired fear extinction. However, the functional cortical regions downstream of the MD remain unknown. Notably, a previous study reported impaired fear extinction in patients with PTSD, showing abnormal activation in the dorsal ACC.^[^ ^38^ ^]^ Furthermore, multiple studies showed a decreased ACC volume in patients with PTSD, which is speculated to interfere with fear extinction.^[^ ^39^, ^40^ ^]^

In the present study, we identified the activated projections from the MD to the ACC during fear extinction using the Fos‐TRAP strategy. Our data unequivocally demonstrated that abnormal connectivity between the MD and ACC contributes to impaired fear extinction in a mouse model of PTSD, which is in agreement with previous clinical findings. Additionally, our results demonstrated that the MD‐ACC PV^+^ circuit was more vulnerable to PTSD‐related stress, potentially because of the tighter connectivity between the MD and ACC PV interneurons. However, we only observed an increased frequency of sIPSCs in pyramidal cells from layer II/III in mice with PTSD, leading to a reduced sEPSC/sIPSC frequency and amplitude ratio. The absence of detectable changes in sEPSCs may be attributed to the following two factors. First, it may be due to the anatomical architecture of the neural circuit. The excessive activation of interneurons directly increases GABA release, which is reflected by the increased frequency of sIPSCs rather than sEPSCs. This increased frequency of sIPSCs may lead to hyperactivity in ACC pyramidal neurons, resulting in the release of excitatory neurotransmitters. However, in Figure 5, we recorded layer II/III pyramidal neurons, which primarily receive excitatory inputs from the thalamus rather than cortico‐cortical sources. Therefore, observing changes in sIPSCs is scientifically relevant. The second reason may be the compensation and homeostatic plasticity. Additionally, homeostatic plasticity may stabilize synaptic strength, counterbalancing the increased excitatory input to GABAergic neurons and maintaining constant sEPSC levels in pyramidal neurons. Our findings echo previous research showing that the MD‐PFC has more connections to PV interneurons than the auditory thalamocortical circuit, suggesting a synaptic sensitivity to stress.^[^ ^41^ ^]^ Another study highlighted that acute foot shock stress activates PV interneurons in the mPFC, leading to decreased mPFC activity, whereas silencing of PV interneurons in the mPFC affects fear extinction retrieval.^[^ ^42^ ^]^ Recent studies in rodent models have established the ACC as a critical neural substrate for the modulation of fear memory.^[^ ^43^, ^44^, ^45^ ^]^ On the one hand, the inactivation of ACC before exposure to fear conditions blocked contextual fear acquisition and did not influence innate fear responses, suggesting the necessity of ACC for the acquisition of contextual fear.^[^ ^44^ ^]^ On the other hand, direct electrical stimulation of the ACC in mice produced fear‐like freezing responses and induced long‐term fear memory, including contextual and auditory fear memory.^[^ ^45^ ^]^ These studies prove that ACC plays an important role in the acquisition and long‐term maintenance of conditioned fear. In addition, ACC is also involved in the process of fear memory extinction.^[^ ^46^ ^]^ ACC is a heterogeneous structure composed of GABAergic interneurons and excitatory pyramidal neurons. Interneurons can maintain local E/I balance by inhibiting neighboring pyramidal neurons. A previous study reported impairment of observational fear due the activation of PV interneurons in the ACC.^[^ ^15^ ^]^ Another study found that selective deletion of Nrxn3 in somatostatin expressing interneurons in ACC significantly increased observational fear.^[^ ^47^ ^]^ Thus, different types of neurons in ACC may play different roles in the formation, maintenance, and extinction of fear memories. In our study, we found delayed fear memory extinction and the E/I imbalance in ACC in PTSD mouse model. This E/I imbalance in the ACC may be mediated by various factors, including the alterations of thalamic input, which can ultimately disrupt the neural circuitry underlying fear extinction. Understanding the specific roles of different neuronal subtypes in the ACC and their contribution to the E/I balance is therefore pivotal for elucidating the mechanisms of delayed fear memory extinction and for developing targeted interventions.

Kv3 channels are voltage‐gated K+ channels that are involved in the rapid repolarization of action potentials in neurons with high‐frequency firing capabilities.^[^ ^23^ ^]^ Both rodents and humans possess four Kv3 genes, namely, Kv3.1, Kv3.2, Kv3.3, and Kv3.4, all of which play a role in multiple psychiatric diseases, such as schizophrenia and Alzheimer's disease.^[^ ^48^, ^49^ ^]^ Furthermore, Kv3 has been reported to regulate fear discrimination by regulating interneuron firing in the underpinning corticolimbic circuitry.^[^ ^50^ ^]^ Ablation of Kv3.1 and Kv3.3 channels has been shown to disrupt thalamocortical oscillations both in vitro and in vivo.^[^ ^51^ ^]^ Despite evidence indicating the influence of Kv3 on thalamocortical circuit function and suggesting that the Kv3 channel is a promising drug target, the current array of specific targeted molecules for therapeutic intervention remains relatively scarce.

Rather than finding novel agonists, we focused on the phosphorylation of the Kv3.2 channel in this study. We observed a significant decrease in the half‐width of neurons in the MD caused by the increased phosphorylation of Kv3.2. Phosphorylation inhibits the activity of Kv3 channels, whereas dephosphorylation increases the Kv3 current and promotes high‐frequency spiking.^[^ ^24^ ^]^ Targeted regulation of key phosphorylated enzymes has the advantages of high selectivity, efficiency, and reversibility, and has been widely used in various pathological processes. Our study found that increased expression of PPP6C, a dephosphorylase of the phosphoprotein phosphatase family,^[^ ^52^ ^]^ enhanced Kv3.2 function in a PTSD mouse model. We observed that PPP6C interacted with Kv3.2, affecting the firing of neurons and connections of the thalamocortical circuit. Therefore, PPP6C may be a potential therapeutic target for the clinical treatment of fear extinction impairment in patients with PTSD.

Given the crucial role of PPP6C in regulating the delicate balance between phosphorylation and dephosphorylation, direct interference at the DNA level using CRISPR technology could disrupt cellular homeostasis. Therefore, we targeted PPP6C at the RNA level, as this approach offers a more precise and controlled means of intervention without compromising the overall cellular equilibrium. Owing to its small size, siRNA has become a leading therapeutic tool for regulating the expression of specific genes. Compared to other technologies like CRISPR, siRNA‐mediated gene silencing is transient, which allows us to study the effects of PPP6C downregulation on Kv3.2 phosphorylation and MD neuron activity in a controlled manner. In addition, siRNA therapy can be designed with high specificity, minimizing the risk of off‐target gene silencing. This is particularly important when studying complex neurological disorders like PTSD, where minimizing off‐target effects is crucial for accurate interpretation of results. Notably, a suitable siRNA drug delivery system is required because of its poor stability in vivo and the difficulty in efficiently entering target cells. LNPs have recently become the leading non‐viral delivery system for the efficient delivery of small‐molecule drugs and nucleic acids.^[^ ^53^ ^]^ Therefore, we constructed PPP6C siRNA‐LNPs to knockdown PPP6C expression in the MD in a mouse model of PTSD, which has been shown to be an efficient and non‐toxic method for delivering siRNA to the brain. A previous study reported that 5 mg ml^−1^ LNP‐siRNA could significantly knock down RNA without causing cytotoxicity or immune stimulation in the brain.^[^ ^54^ ^]^ Moreover, the siRNA‐LNPs immediately acted after injection and lasted for days or longer. For example, patisiran, an FDA‐approved RNA drug, is an siRNA treatment for a genetic disease that can last up to 3 weeks after each injection.^[^ ^55^ ^]^ Notably, lysosome escape is critical for the intracellular stability and effective delivery of siRNA. The ability of LNPs to facilitate lysosomal escape can significantly influence their therapeutic potential. Future studies should focus on how to modify LNP to improve its lysosome escape rate and ultimately improve the delivery effect of LNP. In this study, 1 mg ml^−1^ LNP‐siRNA was injected into the MD, which acted during the first 24 h and could last longer than 72 h.

In summary, we have identified the leading cause of abnormal thalamocortical connections in a mouse model of PTSD. We effectively improved fear extinction in mice with PTSD by administering LNP‐siRNA. Our study provides a new strategy for treating fear extinction by targeting the dephosphorylation of Kv3.2 via siRNA drugs.

## 4 Experimental Section

### Animals

C57BL/6J wild‐type male mice were obtained from the Fourth Military Medical University animal facility. PV‐Cre mice, SST‐Cre mice, and PV‐tdTomato mice were obtained from Jackson Laboratory, USA. The c‐Fos‐CreER^T2^ mouse line^[^ ^56^ ^]^ was obtained from Shanghai Model Organisms (stock no. NM‐ KI‐200110). Mice were housed in a temperature‐controlled environment under a 12 h light/12 h dark cycle and had access to food and water ad libitum. All procedures were approved by the Institutional Animal Care and Use Committee of the Fourth Military Medical University and conformed to the Guide for the Care and Use of Laboratory Animals published by the National Institutes of Health (NIH). Only male adult mice were used in this study and were randomly allocated to different experimental groups.

### Animal Model (SPS&S stress)

As described previously, the PTSD mouse model was established.^[^ ^16^ ^]^ Four stressors: restraint stress, forced swimming, deep anesthesia, and unconditioned foot shock were used. The mouse fetter was used to constrain the experimental groups of mice for 4 h, following which mice were left free in a big cage to rest for 30 min. Subsequently, the mice were forced to swim for 20 min in a plastic tube filled with water. The mice were then exposed to ether and anesthetized until the characteristics of rapid breathing and loss of responses to toe and tail pinch were evident. After 30 min, an unconditioned foot shock (0.8 mA, 5 s) was applied to the experimental group in a chamber with an electrical grid floor to provide foot shock. The mice were then returned to their home cage and left undisturbed.

### Viruses

AAV2/9‐hSyn‐GCaMP6s, AAV2/9‐hSyn‐eNpHR3.0‐EYFP were purchased from Brain VTA, Wuhan, China. AAV2/9‐EF1a‐DIO‐hChR2(H134R)‐EYFP, RV‐EnvA‐ΔG‐eGFP (serotype 1, 1.50 × 10^8^ IFU/ml), AAV2/9‐hSyn‐DIO‐mCherry, AAV2/9‐EF1α‐DIO‐NLS‐mCherry‐F2A‐TVA‐ T2A‐RVG, AAV2/9‐EF1α‐DIO‐oRVG(19G), AAV2/9‐CaMKIIα‐Cre, AAV2/9‐hSyn‐hChR2(H134R)‐EYFP, AAV2/9‐hSyn‐hChR2(H134R)‐mCherry, AAV2/1‐hSyn‐Flp (titer 1.06 × 10^13^ vector genome per ml (vg/ml)), AAV2/9‐hSyn‐Con‐Fon‐hM4D(Gi)‐mCherry, AAV2/9‐hSyn‐Con‐Fon‐mCherry, AAV2/8‐CAG‐pre‐GRASP‐mCerulean, and CAG‐DIO‐post‐GRASP‐P2A‐tdTomato were purchased from Brain Case, China. AAV2/9‐fSST‐mNeonGreen‐CW3SL were purchased from Taitool Bioscience Co., China. The titers of the above viruses were (1–8) × 10^12^ vg ml^−1^ except for individual labeling.

### Surgery and Virus Injection

Mice were anesthetized with isoflurane (4% for induction and 1.5% thereafter for maintenance) and head‐fixed in a surgical stereotactic apparatus. Injections were targeted to MD [−1.70 mm anterior‐posterior (AP), ±0.30 mm medial‐lateral (ML), −3.40 mm dorsal‐ventral (DV)], and ACC (+1.00 mm AP, ±0.35 mm ML, −1.80 mm DV). Standard injection volumes were 100 nl for MD and 300 nl for ACC, which were used in all experiments except the specific retrograde tracing experiments with RV. In the RV tracing experiment, the virus injection for AAV helper and CaMKIIα‐Cre mixture was 300nl, and RV (^△^G) was 80 nl for each mouse. For fiber photometry recordings, 100 nL of AAV2/9‐hSyn‐GCaMP6s was injected into the left MD (−1.70 mm AP, ±0.30 mm ML, −3.40 mm DV) of wild‐type mice, which were implanted with a unilateral optic fiber (200 µm core, 0.39 NA) at the same coordinate. For behavioral manipulation experiments using optogenetics, unilateral optic fiber implants (200 µm core, 0.39 NA) were inserted over the MD (−1.70 mm AP, +0.30 mm ML, −3.40 mm DV) and ACC (+1.00 mm AP, +0.35 mm ML, −1.80 mm DV). The implant was secured to the skull with dental cement. Mice were given sustained‐release buprenorphine (1 mg kg^−1^) as an analgesic after the surgery. Behavioral testing began 3 weeks post‐surgery to allow for the expression of virally delivered proteins.

### Immunohistochemistry

Histological analysis was performed as previously described.^[^ ^13^, ^57^ ^]^ Mice were anesthetized using an overdose of isoflurane and trans‐cardially perfused with 50 ml of 0.01 m phosphate‐buffered saline (PBS) (pH 7.4) followed by 50 ml of 4% paraformaldehyde in 0.2 m phosphate buffer (pH 7.4). Brains were rapidly removed and fixed for 4 h at 4 °C. After dehydrating twice with 30% sucrose solution for 48 h at 4 °C, brain sections (50 µm) were sliced on a freezing microtome CM1950 (Leica, Germany). The brain regions included PoM, FrA, and TRN. The sections from each group (3–6 sections per region) were rinsed in PBS + 0.2% Triton X‐100 (PBS‐T), with 5% normal goat serum for 2 h, followed by incubation with primary antibodies overnight at 4 °C. Sections were then washed 3 times with PBS and probed with secondary antibodies for 4 h. After three more washing steps of 10 min each in PBS, the slices were incubated with 4′,6‐diamidino‐2‐phenylindole (DAPI) for nuclear staining for 15 min. Finally, the slices were washed 3 more times with PBS and mounted on microscope slides. Sections were observed with a confocal microscope (FV3000, Olympus, Japan) and 10× objective fluorescent microscope VS200 (Olympus, Japan). Antibodies used for staining were as follows: primary antibodies: rabbit anti‐Arc antibody (1:3000, Synaptic Systems, Germany), mouse anti‐PV antibody (1:2000, Swant, Switzerland). The corresponding fluorescence secondary antibodies were goat anti‐rabbit Alexa Fluor 488 (1:800, Invitrogen, America), goat anti‐mouse Alexa Fluor 488 (1:800, Invitrogen, America) and DAPI (1:1000, Sigma‐Aldrich, America).

### Fear Extinction Test

Fear conditioning and extinction protocol were performed in two different chambers (Chamber A or B). Chamber A (Vanbi, China) was a square chamber with an electrical grid floor to provide foot shock to the animals, and Chamber B (Vanbi, China) was a plastic tub (20 cm diameter, 30 cm height, rough surface). The mice were put into the experimental environment for 1 h before each behavioral experiment. Chamber A (Vanbi, China) was a square chamber with an electrical grid floor to provide foot shock to the animals, and Chamber B (Vanbi, China) was a plastic tub (20 cm diameter, 30 cm height, rough surface). Twelve days after SPS&S, on day 13, each animal was placed in chamber A for 2 min for habituation. Next, a 30‐s tone referred to CS (75 dB, 4500 Hz) and a foot shock referred to US (0.8 mA, 2 s) were performed in the last 2 s of tone stimulation. After the foot shock, there was a 60 s intertrial interval. CS and US were administered for 10 rounds in each trial. On day 14, the context test was conducted for which animals were first placed in chamber A for a 5 min exploration. After a 2 h break, the cue test continued, and the animals explored chamber B for 3 min and then were subjected to a 3 min conditioned tone (75 dB, 4500 Hz) without any other stimulation. On day 15, each animal was placed in chamber B for fear extinction. After 2 min of habituation, 30 consecutive un‐reinforced 30 s‐long CS presentations, with an interval between CS of 60 s.

### Fiber Photometry Recordings

Fiber photometry recordings were carried out as previously described.^[^ ^13^, ^57^ ^]^ 2–3 weeks after virus injection and fiber optic implantation, mice were connected to the recording equipment to confirm the presence of fluorescence signals. Before each recording session, the implanted optic fiber was connected to the recording setup (Thinker Tech, China) through an external optic fiber. To excite GCaMP6s, a 473 nm light‐emitting diode (LED) was reflected off a dichroic mirror (MD498, Thorlabs) that was focused using a 0.4 numerical aperture (NA) 20× objective lens (Olympus) and coupled to an optical commutator (Doris Lenses). An optic fiber (230 µm optical density, 0.37 NA) guided the light between the commutator and the implanted optic fiber. The laser power at the optic fiber tip was adjusted to 0.01 to 0.02 mW to decrease laser bleaching. Fluorescence was bandpass‐filtered (MF525‐39, Thorlabs), and an amplifier was used to convert the CMOS (complementary metal‐oxide semiconductor) (DCC3240M, Thorlabs) current output to signals, which were further filtered through a low‐pass filter (40‐Hz cutoff). The analog voltage signals were digitalized at 50 Hz and recorded by the multichannel fiber photometry recording system (Thinker Tech, China). Before the behavioral experiment, the animals were allowed to get used to the head‐attached device in the home cage. After confirming the expression, the third day of the fear extinction experiment was initiated.

### Fos‐TRAP Labeling

Animals were anesthetized with isoflurane for stereotaxic injections with meloxicam (1 mg kg^−1^) as an analgesic before incisions. Injections were targeted to MD (−1.70 mm AP, +0.30 mm ML, −3.40 mm DV). AAV‐DIO‐eGFP (100 nl) was injected into MD unilaterally per mouse for a week before stimulation. Fos‐CreER^T2^ mice were used for activity‐dependent labeling experiments. Tamoxifen (Sigma‐Aldrich, America) was dissolved at 20 mg ml^−1^ in corn oil (Sigma‐Aldrich, America) by rotation at room temperature for 4 to 8 h. Tamoxifen (20 mg ml^−1^) was stored up to 24 h at 4 °C before use. All injections were delivered intraperitoneally. For each mouse, optimal activity‐dependent labeling was achieved using a target concentration of 100 to 150 mg kg^−1^. Mice were injected with tamoxifen 23 h before the extinction test. Following fear extinction stimulation, mice remained undisturbed for 2 weeks until they were perfused for histological analyses.

### Electrophysiology

As described previously, a whole‐cell patch‐clamp recording was performed.^[^ ^57^ ^]^ The mice were anesthetized with isoflurane and transcardially perfused with ice‐cold carbonated (95% O_2_, 5% CO_2_) cutting solution containing the following: 115 mm choline chloride, 2.5 mm KCl, 1.25 mm NaH_2_PO_4_, 0.5 mm CaCl_2_, 8 mm MgCl_2_, 26 mm NaHCO_3_, 10 mm D‐(+)‐glucose, 0.1 mm l‐ascorbic acid and 0.4 mm sodium pyruvate (300–305 mOsml^−1^). Coronal slices (300 µm‐thick) containing the ACC (Bregma 1.42 to 0.50 mm, determined by the shapes of lateral ventricles and corpus callosum) were prepared using a vibratome VT1200S (Leica, Germany). Whole‐cell patch‐clamp recordings were made using infrared differential interference contrast visualization at 28–30 °C in artificial cerebral spinal fluid (ACSF) containing the following: 119 mm NaCl, 2.3 mm KCl, 1.0 mm NaH_2_PO_4_, 26 mm NaHCO_3_, 11 mm D‐(+)‐glucose, 1.3 mm MgSO_4_, and 2.5 mm CaCl_2_ (pH7.4, 295–300 mOsml^−1^). Patch pipettes were filled with a solution containing the following: 128 mm potassium gluconate, 10 mm HEPES, 10 mm phosphocreatine sodium salt, 5 mm lidocaine N‐ethyl chloride, 1.1 mm EGTA, 5 mm ATP magnesium salt, and 0.4 mm GTP sodium salt (pH 7.3, 300–305 mOsml^−1^). ACC pyramidal neurons were identified according to the morphological and electrophysiological features under a differential interference contrast microscope. The recordings were obtained using a multiclamp 700B amplifier (Molecular Devices) filtered at 5 kHz and sampled at 20 kHz with a Digidata 1550B. Clampex 10.7 was used for acquisition and analysis.

The cell membrane potential was held at −60 mV for PPR recording, and EPSCs in ACC pyramidal/PV^+^/SST^+^ neurons were evoked by a 2‐ms 473‐nm light stimulation. Paired‐pulse stimuli with an interstimulus interval of 50, 100, and 200 ms were applied. The PPR was calculated as the peak current that responded to the second pulse divided by that to the first pulse.

For Kv3.2 current recording, the cell membrane potential was held at −60 mV and current‐voltage (I/V) relationships were generated over a range of −90 to +50 mV with 10 mV steps. It was reported that low concentrations of TEA can block Kv3 channels in recombinant systems and native neurons.^[^ ^58^ ^]^ This primary voltage command included a prepulse to ‐90 mV (for 200 ms) to remove steady‐state inactivation and a second prepulse to ‐40 mV for 20 ms to inactivate voltage‐gated sodium channels and A‐type potassium currents. Neurons were held at a potential of ‐60 mV and allowed to equilibrate for 5 min before voltage step commands were applied in 10 mV increments.

### Chemogenetic Manipulation

For chemogenetic [i.p., hM4D(Gi) or S5E2‐hM3D(Gq)] neuronal activity manipulation experiments, the second‐generation agonist known as C21 (Tocris, United Kingdom) was used. This agonist in dihydrochloride form was dissolved in sterile saline at 10 mg ml^−1^ and kept as frozen aliquots. For each mouse, optimal chemogenetic activity was achieved using a target concentration of 2 mg kg^−1^ (injected intraperitoneally) 30 min before the behavioral epoch of interest.

### Wireless Optogenetic

A wireless photogenetic device described before was used.^[^ ^19^ ^]^ The device incorporated various functional layers (copper metallization), barrier films (perylene and poly(dimethylsiloxane)), and active components (surface mounted chips and µILEDs) fabricated on a substrate of polyimide (75 µm thickness) in an overall planar geometry to facilitate processing by conventional manufacturing techniques. The electrical interface consisted of a pair of metal lines passing along a serpentine interconnect trace to allow vertical and horizontal freedom of motion relative to a connected circular coil (9.8 mm diameter, copper traces: 8 turns, 60 µm width, 18 µm thickness, and 80 µm spacing) with surface mounted chips for power transfer and control via magnetic coupling to a separate RF transmission loop antenna operating at 13.56 MHz. Here, a capacitor (23 pF) provided impedance matching. A Schottky diode rectified the received RF signals, yielding a current source for the µ‐ILEDs. For optogenetic neuronal activity manipulation experiments, eNpHR 3.0 was activated with a 589 nm laser (5 mW, constant yellow light).

### siRNA Construction

Four siRNA were designed to knock down PPP6C, namely PPP6C‐Mus‐208, PPP6C‐Mus‐320, PPP6C‐Mus‐596, and PPP6C‐Mus‐827 (GenePharma, China). PPP6C‐Mus‐208: sense (5′‐3′): GCUCUUGGAAGAGUCGAAUTT, antisense (5′‐3′): AUUCGACUCUUCCAAGAGCTT. PPP6C‐Mus‐320: sense (5′‐3′): CCUGACACAAACUACAUAUTT, antisense (5′‐3′): AUAUGUAGUUUGUGUCAGGTT. PPP6C‐Mus‐596: sense (5′‐3′): GGCGGUUUAUCUCCUGAUATT, antisense (5′‐3′): UAUCAGGAGAUAAACCGCCTT. PPP6C‐Mus‐827: sense (5′‐3′): CACGAAGGCUAUAAGUUUATT, antisense (5′‐3′): UAAACUUAUAGCCUUCGUGTT.

### Encapsulation of siRNA in LNPs

Following in vitro validation, the most efficient siRNA, PPP6C‐Mus‐827 was selected, which was modified with 5′FAM (a green fluorescence) to observe its expression. The transfection efficiency of PPP6C siRNA in vivo was improved by modifying with 2′‐OMe. For the production of siRNA‐loaded LNPs, the method reported previously was followed which employed a microfluidic mixing technique.^[^ ^59^ ^]^ This method involves the rapid mixing of an ethanol phase, which contains the lipid components, with an aqueous phase that includes the siRNA. The lipids used in the formation of LNPs include an ionizable lipid, DSPC (1,2‐distearoyl‐sn‐glycero‐3‐phosphocholine), cholesterol, and a PEGylated lipid. In the ethanol phase, the ionizable lipid was uncharged due to the absence of counterions. Upon mixing with the siRNA‐containing aqueous phase at a low pH, the ionizable lipid becomes protonated and positively charged, facilitating electrostatic interactions with the negatively charged siRNA, leading to the encapsulation of siRNA within the forming LNP. The process was driven by the rapid shift from an ethanol‐rich to an aqueous environment, causing the lipids to become insoluble and assemble into nanoparticles. The ionizable nature of the lipid allows for the siRNA to be tightly bound within the core of the LNP. Following the initial formation, the LNPs undergo a series of pH adjustments, typically through dialysis or tangential flow filtration, to achieve physiological pH, which stabilizes the LNPs and prepares them for biological applications. The lipid composition used was 8‐[(2‐hydroxyethyl) [6‐oxo‐6‐(undecyloxy) hexyl] amino]‐octanoic acid, 1‐octylnonyl ester (SM‐102) /distearoylphosphatidylcholine (DSPC)/cholesterol/α‐[2‐(ditetradecylamino)−2‐oxoethyl]‐ω‐methoxy‐poly(oxy‐1,2‐ethanediyl) (ALC‐0159) (structure shown in Figure 1A) in the molar percentage ratios 50/10/38.5/1.5 (APExBIO, America). Among them, the most critical component was ionizable lipids, considered decisive factors in the delivery and transfection efficiency of siRNA. SM‐102 was used as the ionizable lipid, which could bind to negatively charged siRNA and improve its stability. DSPC, generally saturated phospholipids, could increase the phase transition temperature of cationic liposomes, support the formation of the bilayer structure of layered lipids, and stabilize its structural arrangement. Cholesterol, with strong membrane fusion properties, promoted siRNA uptake and cytoplasmic entry. ALC‐0519, a PEG lipid located on the surface of lipid nanoparticles, improved their hydrophilicity, avoiding rapid clearance by the immune system, preventing particle aggregation, and increasing stability.

### Tissue Lysis, Immunoblots, and Co‐Immunoprecipitations

For immunoblotting analysis, mouse brain tissue samples were homogenized in RIPA lysis buffer containing a protease inhibitor cocktail (Roche Complete) and phosphatase inhibitors (Roche PhosSTOP) on ice for 20 min. After centrifugation at 4 °C and 12 000 rpm for 10 min, the supernatant was collected for protein quantification using BCA Protein Assay Kit (Thermo Scientific) and subsequent protein sample preparation. Analysis was then performed, ensuring equal amounts of total proteins were loaded per lane for immunoblotting. Following gel electrophoresis and transfer onto membranes, primary antibody incubation followed by secondary antibody incubation against the target protein were conducted sequentially. Finally, protein bands were visualized using a chemiluminescence imaging system (Bio‐rad).

For the immunoprecipitation experiment, mouse brain tissue was obtained and added IP lysis buffer (50 mm Tris‐HCl PH 7.5, 1 mm EDTA, 1% TritonX‐100, 150 mm NaCl, 2 mm DTT, 100 µm PMSF) containing preloaded Roche Complete Protease Inhibitor Cocktail and Roche PhosSTOP Phosphatase Inhibitor Cocktail. The brain tissue was homogenized on ice and then incubated at 4 °C for 4 h on a shaker. After centrifugation at 12000 rpm for 15 min, the supernatant was mixed with either anti‐Kv3.2 antibody or normal IgG antibody (2 µg) and incubated overnight on a shaker at 4 °C. Protein A/G Magnetic Beads (MCE, America) were washed 3 times with IP lysis buffer, added to the samples, and then incubated for 4 h on a shaker at 4 °C. Finally, the samples were washed 3 times with the IP lysis buffer, loading buffer was added and loaded onto SDS‐PAGE gels.

### RNA Extraction and RT–qPCR

The brain tissue was lysed using TRIZOL, followed by adding chloroform and incubating on ice. After centrifugation, the supernatant was collected, mixed with isopropanol, and incubated for 20 min. Then, the supernatant was discarded after centrifugation, and the RNA pellet was air‐dried in a laminar flow hood and dissolved in an appropriate amount of DEPC‐treated water. For reverse transcription, 1 µg of total RNA was used with the RevertAid First Strand cDNA Synthesis Kit (Thermo Scientific, USA). Real‐time qPCR reactions were performed using AceQ Universal SYBR qPCR Master Mix (Vazyme, USA) in a final volume of 20 µl. The relative gene expression levels were quantified using cyclophilin as a housekeeping gene (2[Ct cyclophilin − Ct target gene]).

### Cell Counting

Brain sections were imaged with a 10× magnification objective on a confocal microscope FV3000 (Olympus, Japan) or fluorescence microscope VS200 (Olympus, Japan). Images were processed using Image J, and quantifications were performed manually from 3–5 sections per animal. All counting experiments were conducted blinded to the identity of experimental groups.

### Image Analysis

To analyze synapses labeled by mGRASP, ACC‐containing sections were imaged using a confocal microscope FV3000 (Olympus, Japan). Multiple optical sections (0.5 mm thickness) were imaged to cover the entire z‐axis of the section. Thereafter images were reconstructed in 3D and analyzed using Imaris Image analysis software (Imaris 9.0.0, Oxford Instruments). 3D isosurfaces were created for each PV^+^, SST^+^, or CaMKII^+^ neuron identified by the tdTomato signal expressed in post‐mGRASP neurons and were masked to isolate the fluorescent signals within and surrounding the cell body. For each masked cell, a second round of 3D isosurfaces was created for the mGRASP signal to form a mask around all mGRASP labeled synapses. To avoid false‐positive counting of synapses, it was visually confirmed that the synapses were formed on postsynaptic cortical neurons with thalamic axons identified with the pre mGRASP mCerulean signal. Care was taken to ensure that the entire mGRASP signal was covered by the isosurfaces created. The number of such isosurfaces created was used to quantify the number of synapses per cell, while their volumes were used to quantify the volume of the synapses.

### Behavior Analysis

*Pose Estimation*: The activity state of mice was examined by using a camera to record their movements from a bird's eye view at a frame rate of 25. DeepLabCut was employed for unmarked pose estimation of the mice and focused on tracking four distinct points on the body of each mouse – the snout, left ear, right ear, and tail base. These points were chosen as they best represent the overall activity state of the mice. Twelve hundred frames extracted from 6 different videos were labeled and trained a Resnet_50 neural network model over 500000 iterations for training purposes. The resulting model proved highly effective and was subsequently used to predict activities across all 11 videos.

*Machine Learning Models*: A comprehensive data were undertook preprocessing on the information obtained from pose estimation and fiber recording, leveraging Python as the principal instrument. The code was available on GitHub (https://github.com/GUO‐neuroLAB/Code_available.git). Subsequently, a diverse range of machine learning models was designed, including the random forest model, polynomial regression model, and support vector machine model.

1) Data Preprocessing: To ascertain if the mice were in a state of freezing, their speed was measured at every given moment by utilizing the spatiotemporal position information derived from DeepLabCut. An average of the speeds corresponding to various feature points of the mice was then calculated and smoothed out the speed data (with a window set at 6). A speed threshold was established at 2 (Pixel/Frame), and any value below this was interpreted as the mice being in a state of freezing. This conclusion was drawn based on comparative analysis with original data sets. For fiber recording data, delta F/F, cumulative delta F/F, rate of change concerning delta F/F, maximum delta F/F, and time taken to achieve maximum delta F/F was calculated.
2) Random forest model: A random forest model was used to determine whether MD activity was significantly involved in fear behavior in mice and understand the correlation between the MD calcium signals and the respective behaviors in mice at each given moment. A total of 204255 data points was segmented into an 80% training set and a 20% test set. The random forest model was trained with specific parameters, including n_estimators = 100 and max_depth = 5, to circumvent overfitting through a rigorous process of 5‐fold cross‐validation. Besides this primary model, a control model was also constructed where the label set was shuffled for comparison. The predictive performance of the model was then evaluated by calculating its accuracy level using various metrics, such as the confusion matrix, ROC curve, and AUC.
3) Polynomial regression model: To better understand whether the MD activity could quantitatively represent fear behavior in mice, a polynomial regression model was constructed and utilized. This model was designed to train on various factors, including the calcium signals from the MD, specific conditions of mice, the trails they were navigating, and their behavior during each trial. From a total of 255 data points collected for this study, they were strategically partitioned into two distinct sets – 80% was allocated for training, while the remaining 20% was reserved for testing purposes. This approach ensured that the model had ample data to learn from and enough data left over for validation. The polynomial regression model was meticulously trained with set parameters of degree = 2 using a method known as five‐fold cross‐validation to prevent potential overfitting issues that could distort the results. As part of the robust analysis process, a control model was also developed by randomly shuffling the label set. An error threshold was established at six units and assessed the predictive performance of the model by determining both the number and percentage of successful predictions made. This comprehensive approach allowed to fully evaluate how accurately the polynomial regression model (equation shown below) could predict fear behaviors in mice based on their MD activity.
(1) $$y=\beta_{0}+\sum_{i=1}^{k}\beta_{i}x_{i}+\sum_{i=1}^{k}\sum_{j=1}^{k}\gamma_{\mathrm{ij}}x_{i}^{\alpha_{i}}x_{j}^{\alpha_{j}}$$
4) Support vector machine model: A support vector machine model was also employed to delve deeper into the potential correlation between MD activity and mice fear behavior. This model was trained utilizing the calcium signals from the MDs of mice, their navigational trails, behavioral patterns, and their condition. A comprehensive dataset comprising 255 data points was methodically divided into an 80% training set and a 20% test set. The support vector machine model was trained using parameters, such as “C”: 0.1, “gamma”: 0.1, “kernel”: “linear”, to circumvent the risk of overfitting; this process was facilitated through five‐fold cross‐validation. Additionally, a control model was established by randomly shuffling the label set. The efficacy of the predictive capabilities of the model was assessed through metrics including accuracy rates, confusion matrix analysis, ROC curve plotting, and Area Under Curve (AUC) calculations.

### Statistical Analyses

Data were analyzed using GraphPad Prism, version 8.0 (GraphPad Software), and SPSS v26.0. The normality and the homogeneity of variance tests were performed with the Shapiro‐Wilk test and Levene's test, respectively. Data that met these two conditions were analyzed using a two‐tailed, unpaired or paired t‐test, a two‐way ANOVA, or a repeated‐measures ANOVA. Data sets that were not normally distributed were analyzed with a nonparametric test. Data were reported as the mean ± SEM. Details of particular statistical analyses are presented in Table S1 (Supporting Information). A *P*‐value of less than 0.05 was considered statistically significant.

### Ethical Statement

Animal experiments were performed under a project license (FMMULL‐220926) granted by the Institutional Animal Care and Use Committee of the Fourth Military Medical University, in compliance with the Guide for the Care and Use of Laboratory Animals published by the National Institutes of Health.

## Conflict of Interest

The authors declare no conflict of interest.

## Supporting information

Supporting Information

Supplemental Table 1

Supplemental Table 2

Supplemental Table 3

## Data Availability

The data that support the findings of this study are available from the corresponding author upon reasonable request. The code generated in this study is available on GitHub (https://github.com/GUO‐neuroLAB/Code_available.git).
