# Supplementary material for: Restoring the Function of Thalamocortical Circuit Through Correcting Thalamic Kv3.2 Channelopathy Normalizes Fear Extinction Impairments in a PTSD Mouse Model
Source: Adv Sci (Weinh). 2023 Dec 16;11(9):2305939. doi: 10.1002/advs.202305939 (PMC10916658; doi:10.1002/advs.202305939)
Supplement: Supplementary file 1 — Supporting Information [file ADVS-11-2305939-s003.pdf]

ADVANCED SCIENCE
Open Access

Supporting Information

for *Adv. Sci.*, DOI 10.1002/advs.202305939

Restoring the Function of Thalamocortical Circuit Through Correcting Thalamic Kv3.2 Channelopathy Normalizes Fear Extinction Impairments in a PTSD Mouse Model

Haoxiang Xiao, Kaiwen Xi, Kaifang Wang, Yongsheng Zhou, Baowen Dong, Jinyi Xie, Yuqiao Xie, Haifeng Zhang, Guaiguai Ma, Wenting Wang, Dayun Feng, Baolin Guo* and Shengxi Wu**

Supporting Information

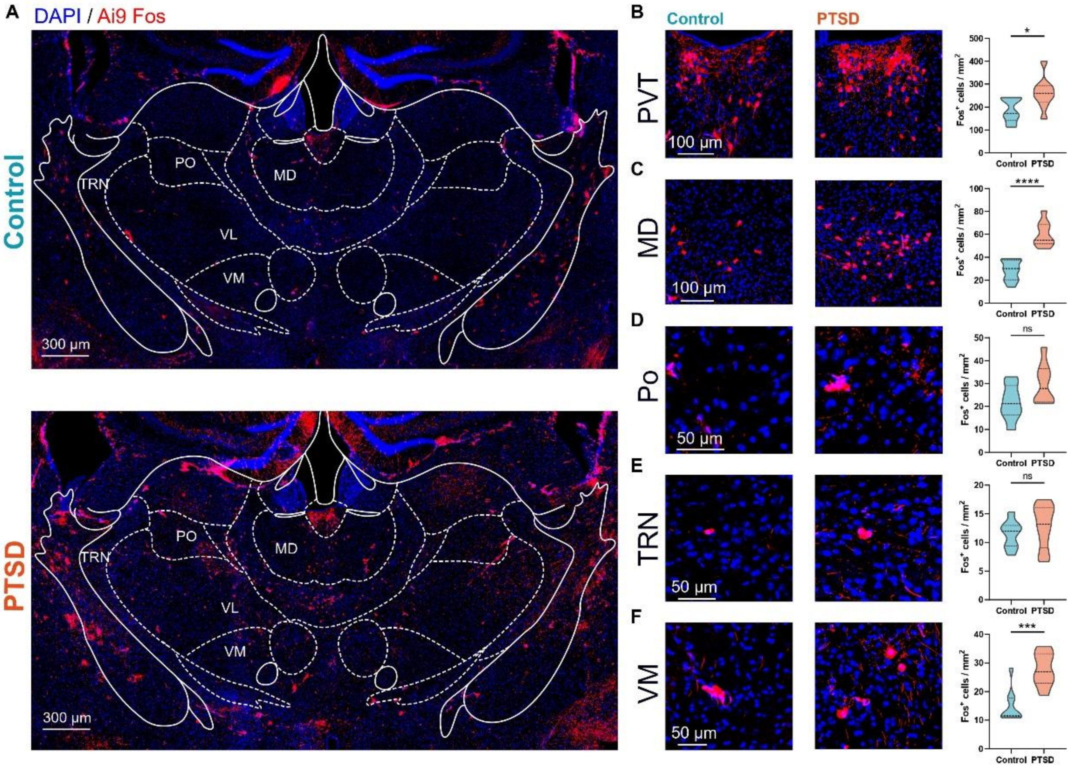

Figure S1. Fos^{+} cell distributions of multiple thalami during extinction in control and PTSD mice. (**A**). Schematic of Fos distributions in multiple thalami in control and PTSD mice; scale bar: 300 μm. (**B-F**) Representative images and quantification of Fos expression in paraventricular nucleus of the thalamus (PVT), mediodorsal thalamus (MD), thalamic posterior (PO), thalamic reticular nucleus (TRN), and ventromedial thalamus (VM) between control and PTSD mice; scale bar: 100 μm **(B** and **C**), 50 μm (**D-F**) (n = 8–9 slices from three mice per group, error bar: mean with SEM, $*P_{\mathrm{PVT}} = 0.0206$, $****P_{\mathrm{MD}} < 0.0001$, $***P_{\mathrm{VM}} = 0.0002$ by two-tailed unpaired Student's t-test). Statistical details are presented in Table S1.

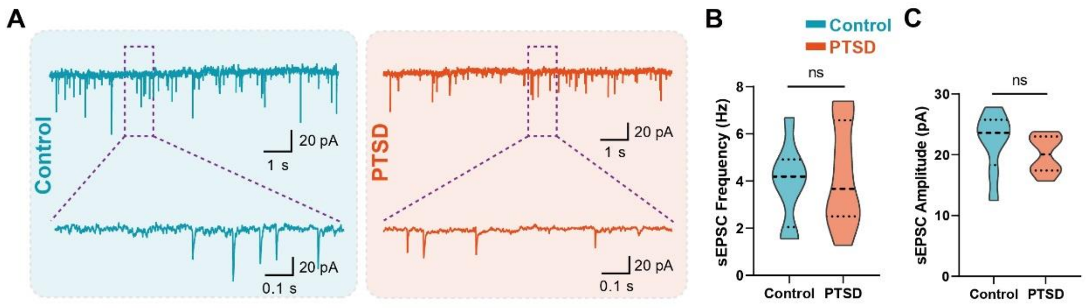

Figure S2. The sEPSC of MD neurons in control and PTSD mice. (**A-C**). Representative traces (**A**) and quantification of sEPSC frequency (**B**) and amplitude (**C**) showing no significant differences in frequency and amplitude between the control and PTSD groups (n = 8 neurons from three mice per group, error bar: mean with SEM, ns_B = 0.7798, ns_C = 0.3253 by two-tailed unpaired Student's t-test). Statistical details are presented in Table S1.

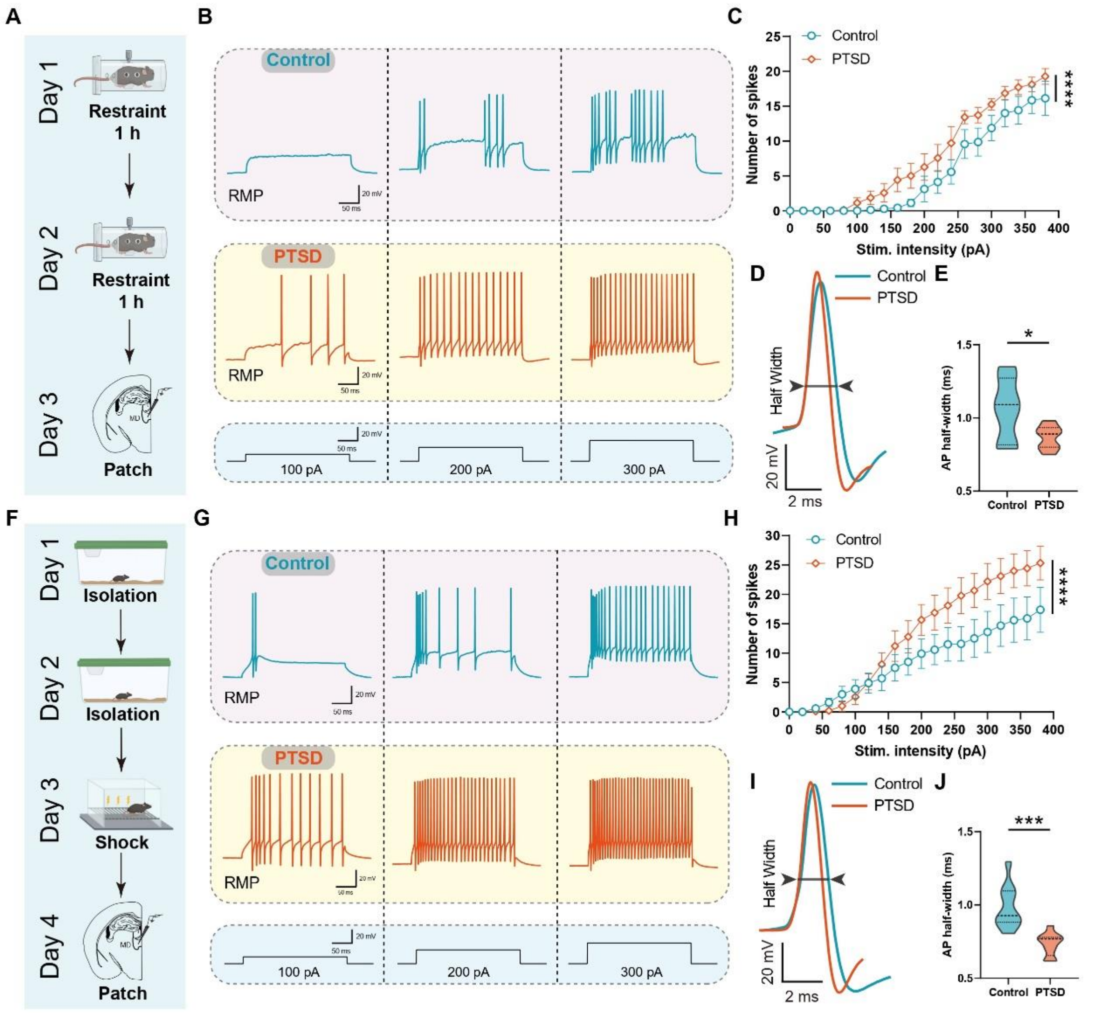

Figure S3. Excitability of MD in two PTSD models. (**A**). Schematic of the restraint stress model. (**B**). Firing of MD neurons in control mice and mice with PTSD after stimulation with different current intensities under current clamp. (**C**). Average number of spikes of MD neurons between control mice and mice with PTSD under current clamp (n = 7 neurons from three mice per group, error bar: mean with SEM, ****$P < 0.0001$ by Friedman's M test with Nimenyi post hoc test). (**D**). Representative image showing the half-width of MD neurons in control mice and mice with PTSD. (**E**). Whole-cell current clamp showing a lower half-width of MD neurons in mice with PTSD than that in control in an acute slice preparation (n = 7 neurons from three mice

per group, error bar: mean with SEM, **P* = 0.0439 by two-tailed unpaired Student's t-test). (**F**). Schematic of the footshock model. (**G**). Firing of MD neurons in control mice and mice with PTSD after stimulation with different current intensities under current clamp. (**H**). Average number of spikes of MD neurons between control mice and mice with PTSD under current clamp (n = 9–10 neurons from three mice per group, error bar: mean with SEM, *****P* < 0.0001 by Friedman's M test with Nimenyi post hoc test). (**I**). Representative image showing the half-width of MD neurons in control mice and mice with PTSD. (**J**). Whole-cell current clamp showing a lower half-width of MD neurons in mice with PTSD than that in control in an acute slice preparation (n = 9–10 neurons from three mice per group, error bar: mean with SEM, ****P* = 0.0003 by two-tailed unpaired Student's t-test). Statistical details are presented in Table S1.

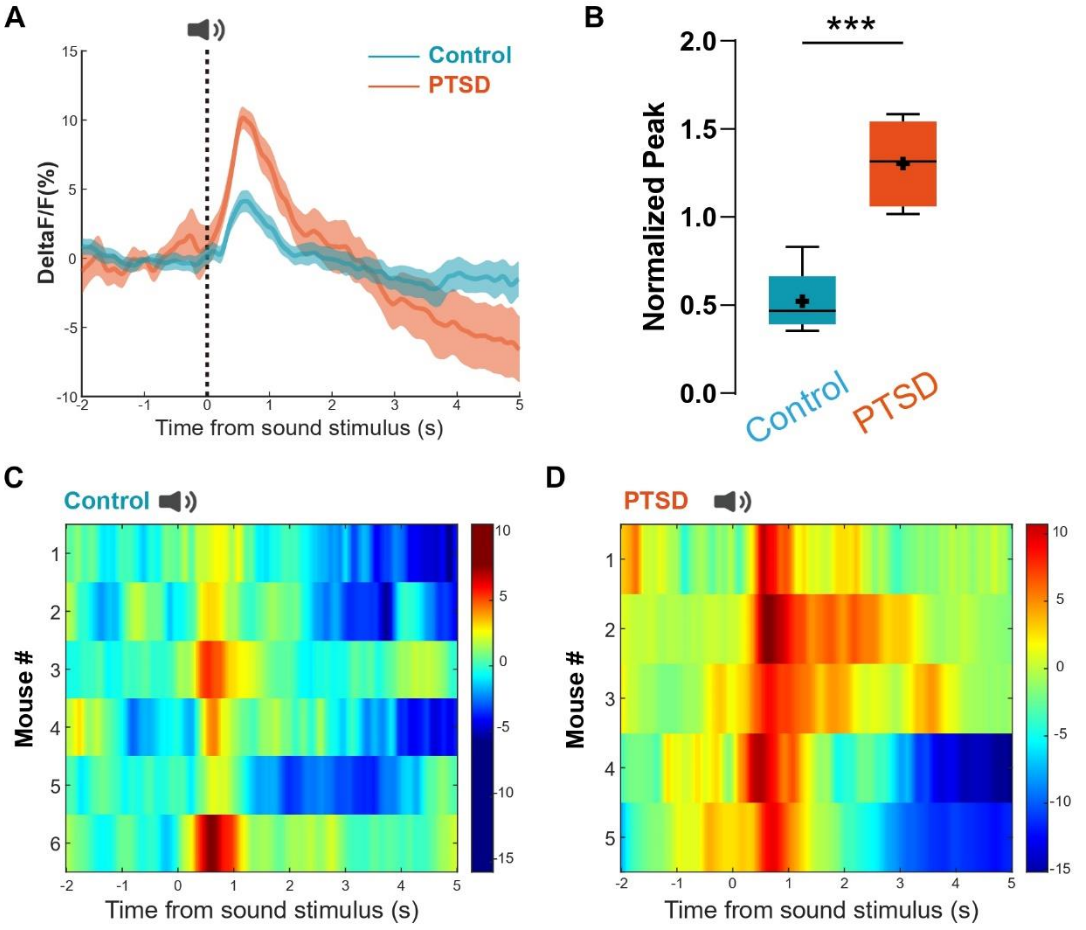

Figure S4. The overactivated response of MD caused by fear-inducing stimuli. (**A**). Temporal progression of MD neuron calcium signals. (**B**). Comparative bar graph of peak MD neuron calcium signals. (n = 5–6 mice per group, error bar: mean with SEM, ***P = 0.0002 by two-tailed unpaired Student's t-test). (**C** and **D**). Heatmap visualization of the MD neuron activity over time. Statistical details are presented in Table S1.

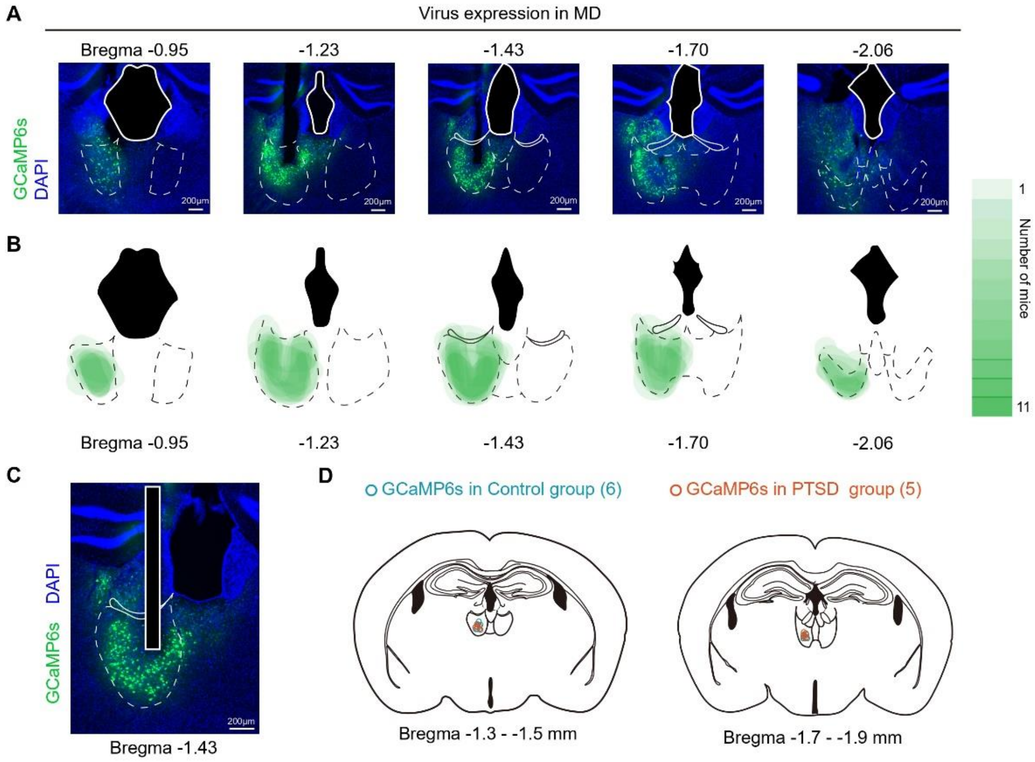

Figure S5. Expression of AAV-hSyn-GCaMP6s-EYFP in MD and implantation of optical fiber in MD (related to Figure 2). (**A-B**). Representative images (**A**) and overlay (**B**) of GCaMP6s-EYFP expression across MD (bregma -0.95 to -2.06 mm). Scale bar, 200 μm (**C and D**). Representative images (**C**, shading: fiber tack) and overlay (**D**) of locations of tips of optical fibers in MD of GCaMP6S control and PTSD groups. Scale bar, 200 μm. n = 6 mice in control group, n = 5 mice in PTSD group.

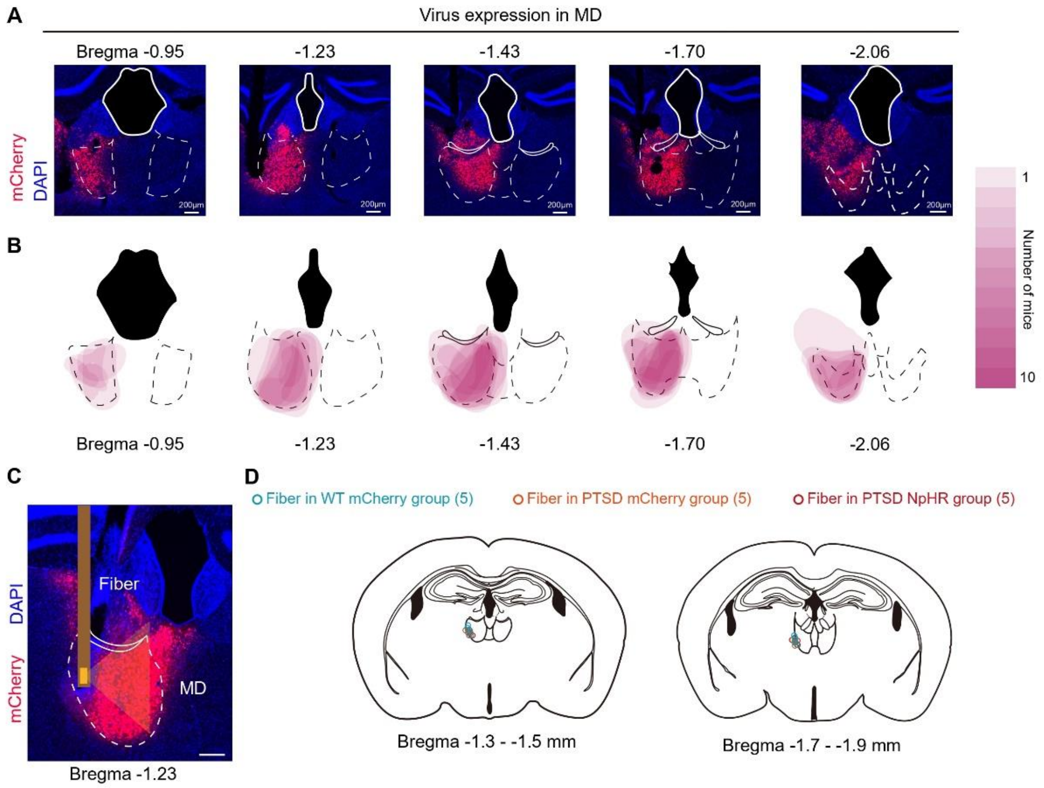

Figure S6. Expression of AAV-hSyn-NpHR-EYFP/mCherry in MD and implantation of optical fiber in MD (related to Figure 3). (**A-B**). Representative images (**A**) and overlay (**B**) of mCherry expression across MD (bregma -0.95 to -2.06 mm). Scale bar, 200 µm (**C and D**). Representative images (**C**, shading: fiber tack) and overlay (**D**) of locations of tips of optical fibers in MD of WT mCherry control, PTSD mCherry control, and PTSD NpHR groups. Scale bar, 200 µm. n = 5 mice per group.

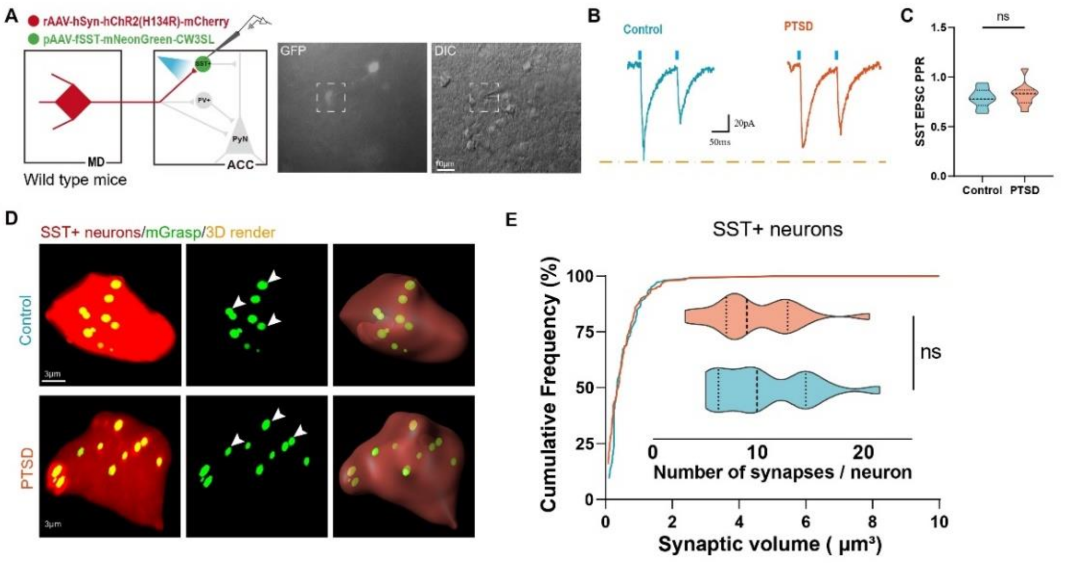

Figure S7. Changes in synaptic connections of SST^{+} neurons in ACC projecting from MD. (**A**). Schematic of whole-cell patch in ACC. Scale bar, 10 μm. Representative paired-pulse traces (**B**) and quantitative summary (**C**) of SST^{+} neurons showing reduced PPR in PTSD mice (n = 9 neurons from three mice per group, error bar: mean with SEM, ns = 0.4930 by two-tailed unpaired Student's t-test). (**D**). High magnification confocal image (left) and 3D renders (middle and right) of a post-mGRASP labeled SST^{+} neuron (red) in the ACC merged with synaptic labeling by mGRASP (green). Arrowheads indicate putative synapses. Scale bars, 3μm. (**E**). Quantification of thalamocortical synaptic frequency in WT mice shows that the SST^{+} neurons in ACC had no significant difference in the frequency of thalamocortical synapses in control and PTSD mice (n = 20–21 neurons from three mice per group, error bar: mean with SEM, ns_{TOP} = 0.5438 by two-tailed unpaired Student's t-test, ns_{Bottom} > 0.9999 by Kolmogorov-Smirnov D test). Cumulative frequency distribution of synapses by volume in SST^{+} neurons in ACC shows no significant difference

between the thalamocortical synapses in control and PTSD mice. Statistical details are in Table S1.

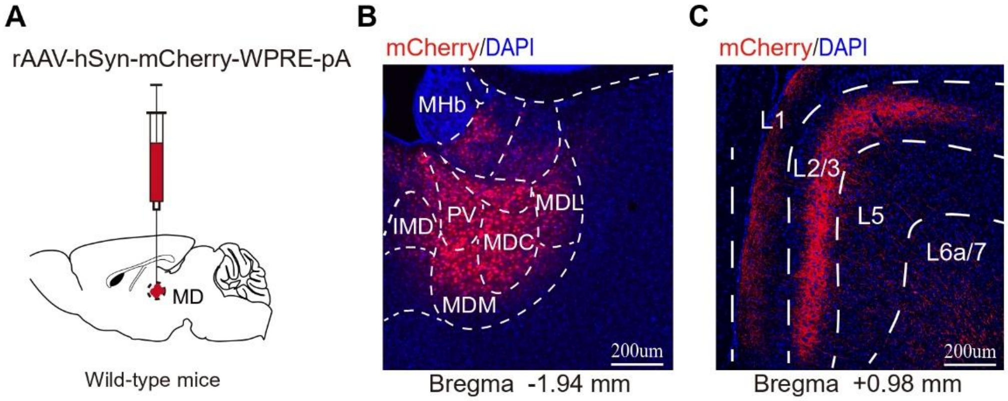

Figure S8. Anterograde tracking of MD brain region. (**A**). Schematic of virus injection (**B**). Representative images of mCherry expression across MD. Scale bar, 200 μm. (**C**). Representative images of MD axons across ACC. Scale bar, 200 μm.

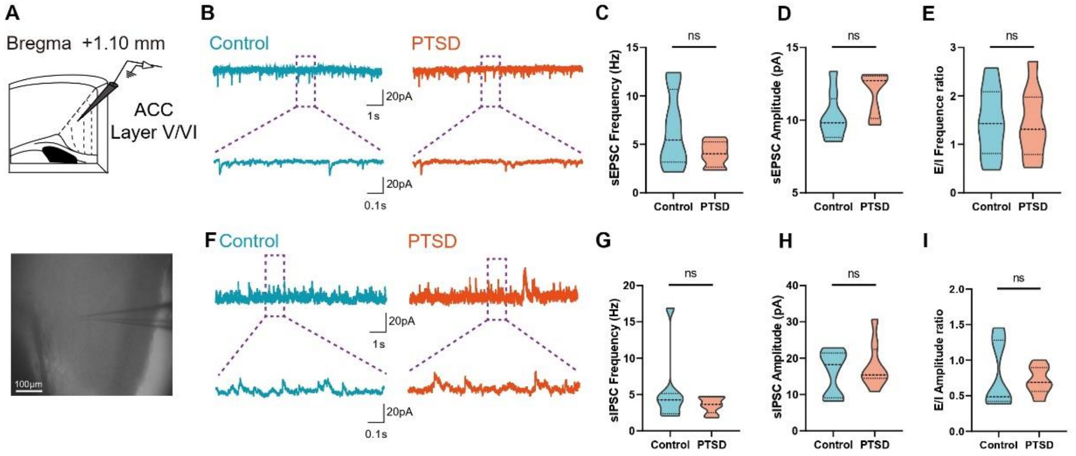

Figure S9. E/I of pyramidal neurons in the ACC layer V/VI between control and PTSD mice. (**A**). Schematic of whole-cell patch in ACC layer V/VI. Bregma = +1.10 mm. Scale bar, 100 μm (**B-D**). Representative traces (**B**) and quantification of sEPSC frequency (**C**) and amplitude (**D**) showing no significant difference in frequency and amplitude between the control and PTSD groups. (**F-H**). Representative traces (**F**), quantification of sIPSC frequency (**G**), and amplitude (**H**) shows no significant difference in frequency and amplitude between the control and PTSD groups (n = 8 neurons from 4–5 mice per group). Quantification of the E/I frequency ratio (**E**) and E/I amplitude ratio (**I**) shows no significant difference between the control and PTSD groups. n = 8 neurons from four to five mice per group, error bar: mean with SEM, ns_C= 0.1151, ns_E = 0.9575, ns_H = 0.5651 by two-tailed unpaired Student's t-test, ns_D = 0.0830, ns_G = 0.7209, ns_I = 0.6454 by Mann-Whitney U test. Statistical details are in Table S1.

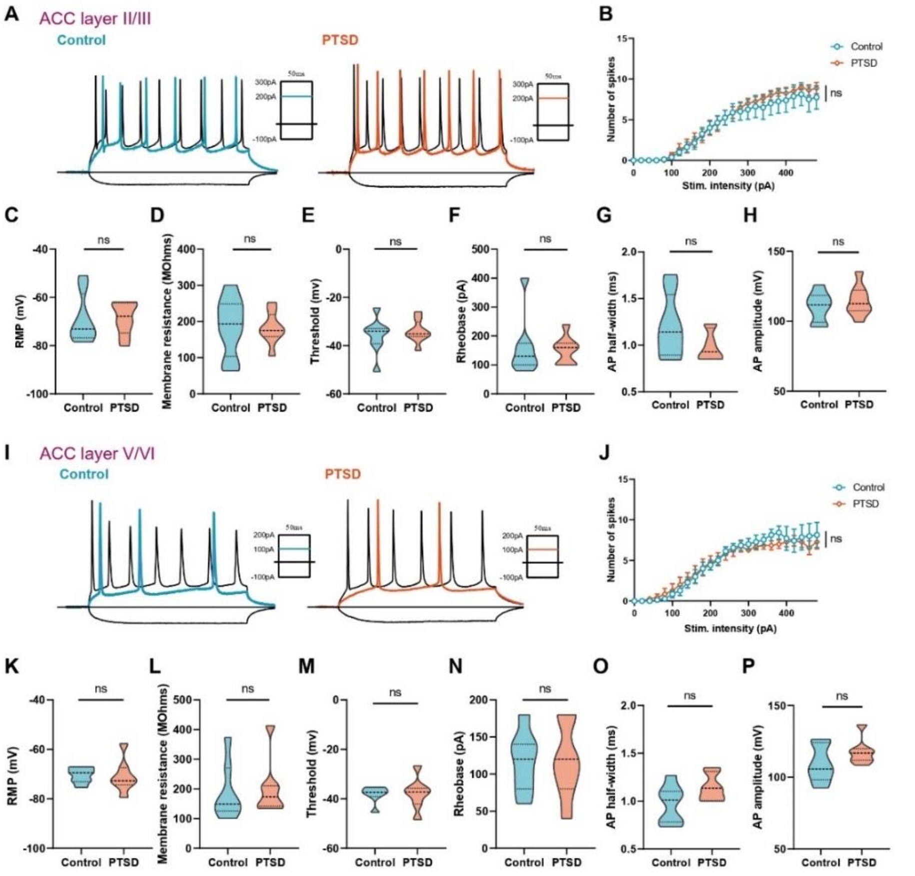

Figure S10. Action potential and active and passive properties of pyramidal neurons in ACC. (**A and I**). APs induced by current injection at -100 pA, 0 pA, 200 pA, and 300 pA in pyramidal neurons in ACC layers II/III and V/VI. (**B and J**). Average number of spikes of pyramidal neurons in ACC between control and PTSD mice under current clamp. (**C-H, K-P**). Quantification of active and passive properties of pyramidal neurons in ACC layers II/III (**C-H**) and V/VI (**K-P**). n = 7–8 neurons from three to four mice per group, error bar: mean with SEM, $ns_B = 0.931$, $ns_J = 0.051$ by two-way ANOVA with Tukey's post hoc test, $ns_C = 0.9760$, $ns_D = 0.8852$, $ns_E = 0.5930$, $ns_H = 0.4493$, $ns_K = 0.9230$, $ns_L = 0.5350$, $ns_N > 0.9999$, $ns_O = 0.0850$, $ns_P = 0.1850$ by two-tailed unpaired Student's t-test, $ns_F = 0.7075$, $ns_G = 0.2239$, $ns_M > 0.9999$ by Mann-Whitney U test. Statistical details are in Table S1.

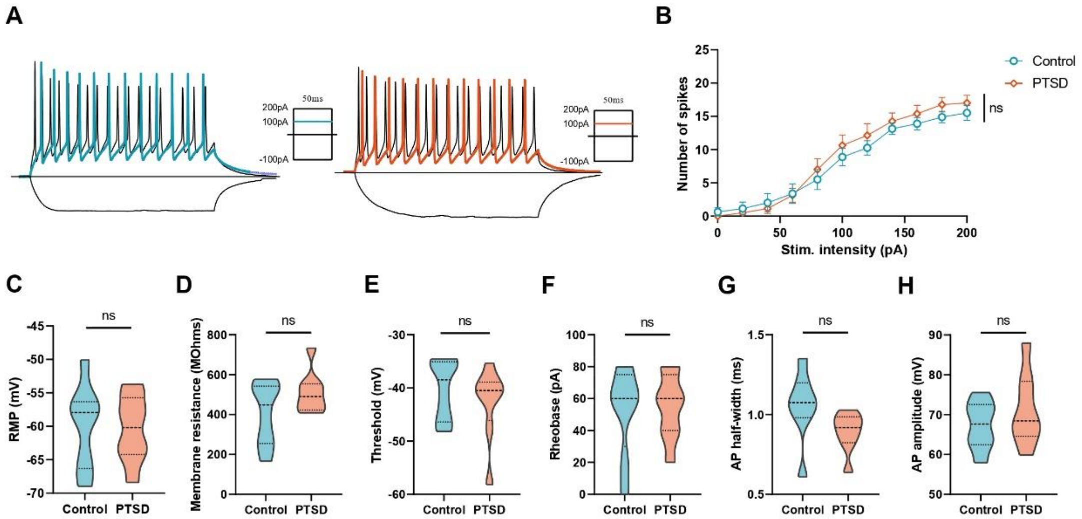

Figure S11. Action potential and active and passive properties of SST+ neurons in ACC. (**A**). APs induced by current injection at -100 pA, 0 pA, 200 pA, and 300 pA in SST+ neurons in ACC layer II/III. (**B**). Average number of spikes of SST+ neurons in ACC between control and PTSD mice under current clamp. (**C-H**). Quantification of active and passive properties of SST+ neurons in ACC layer II/III. n = 8 neurons from four mice per group, error bar: mean with SEM, $ns_B = 0.6095$ by two-way ANOVA with Tukey's post hoc test, $ns_C = 0.8645$, $ns_D = 0.1430$, $ns_G = 0.0820$, $ns_H = 0.3307$ by two-tailed unpaired Student's t-test, $ns_E = 0.5737$, $ns_F > 0.9999$ by Mann-Whitney U test. Statistical details are in Table S1.

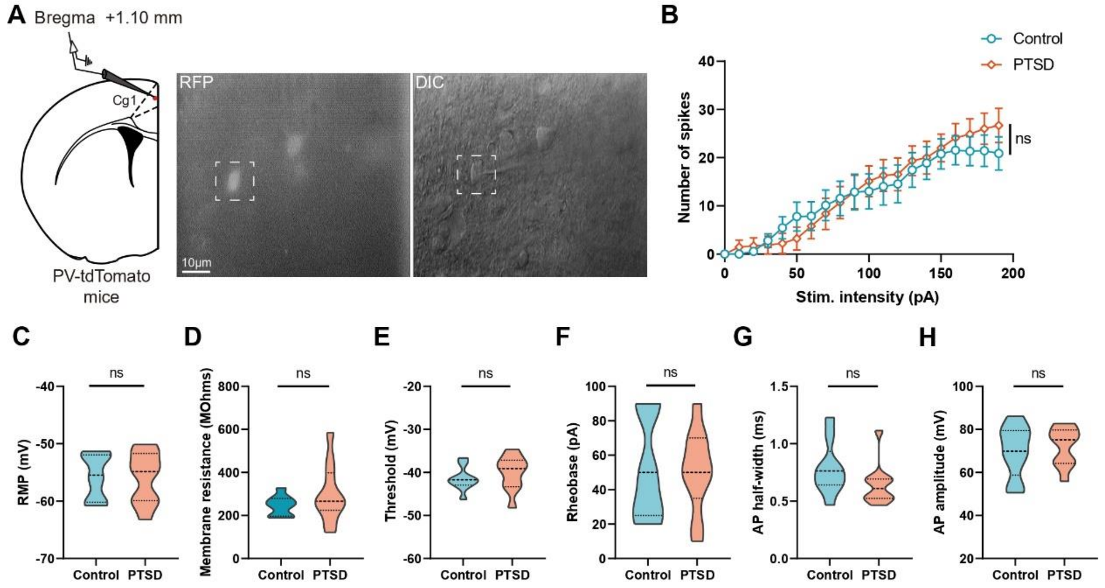

Figure S12. Action potential and active and passive properties of PV⁺ neurons in ACC. (**A**). Schematic of whole-cell patch in ACC (**B**). Average number of spikes of PV^+ neurons in ACC between control and PTSD mice under current clamp (**C-H**). Quantification of active and passive properties of PV^+ neurons in ACC layer II/III. n = 8 neurons from four mice per group, error bar: mean with SEM, $ns_B = 0.4094$ by two-way ANOVA with Tukey's post hoc test, $ns_C= 0.7412$, $ns_D = 0.3212$, $ns_E = 0.0595$, $ns_F = 0.7529$, $ns_G = 0.1087$, $ns_H = 0.8537$ by two-tailed unpaired Student's t-test. Statistical details are in Table S1.

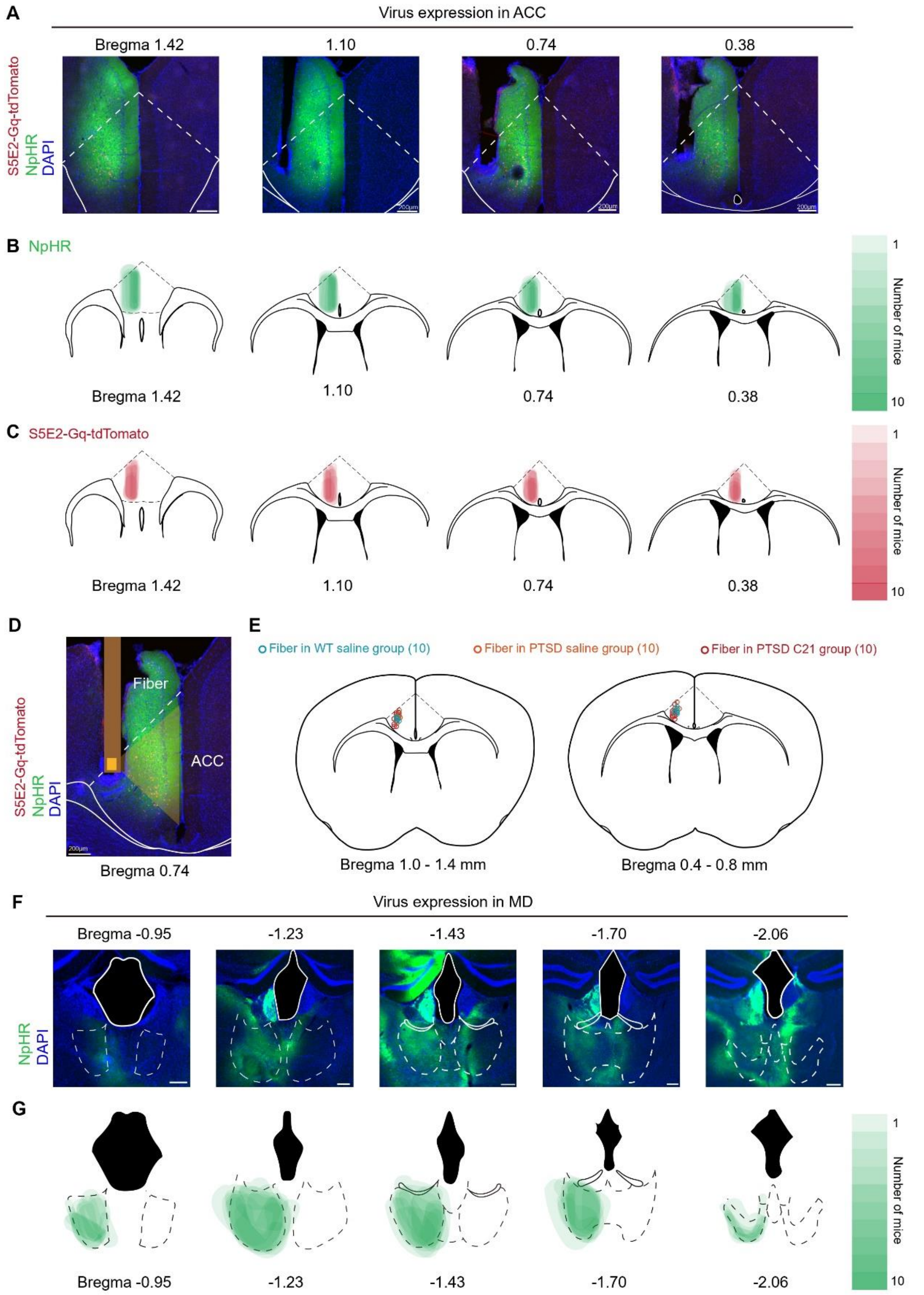

Figure S13. Expression of AAV-S5E2-Gq-tdTomato/AAV-hSyn-NpHR-EYFP in ACC and AAV-hSyn-NpHR-EYFP in MD (related to Fig. 6). (**A-C**). Representative images (**A**) and overlay (**B and C**) of NpHR-EYFP and S5E2-Gq-tdTomato expression

across ACC (bregma 0.38 to 1.42 mm). Scale bar, 200 µm. (**D and E**). Representative images (**D**, shading: fiber tack) and overlay (**E**) of tip locations of optical fibers in ACC of PTSD EYFP Gq saline, PTSD NpHR Gq saline, and PTSD NpHR Gq C21 groups. Scale bar, 200 µm. (**F-G**). Representative images (**F**) and overlay (**G**) of NpHR-EYFP across MD (bregma -0.95 to -2.06 mm). Scale bar, 200 µm. n = 10 mice per group.

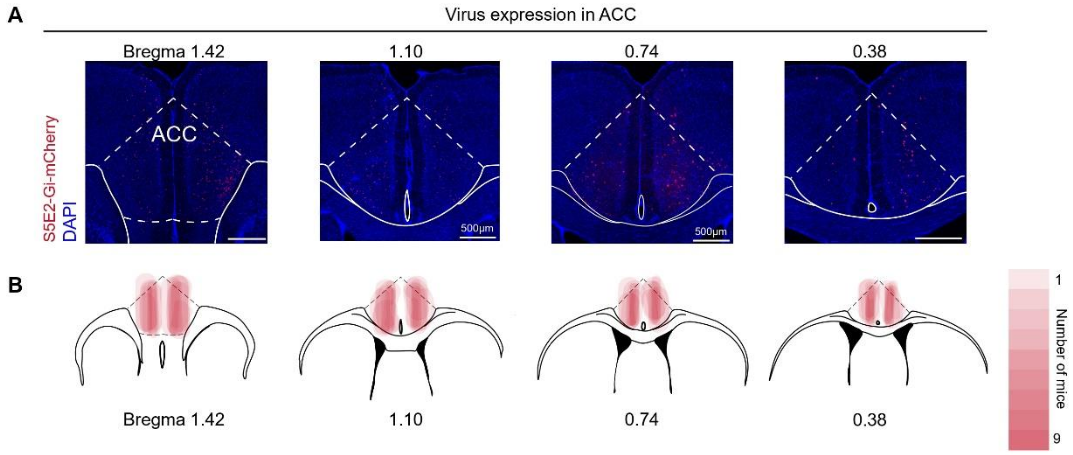

Figure S14. Expression of AAV-S5E2-Gi-mCherry in ACC (related to Fig. 6). (**A-B**). Representative images (**A**) and overlay (**B**) of S5E2-Gi-mCherry expression across ACC (bregma 0.38 to 1.42 mm, n = 9 mice). Scale bar, 200 μm.

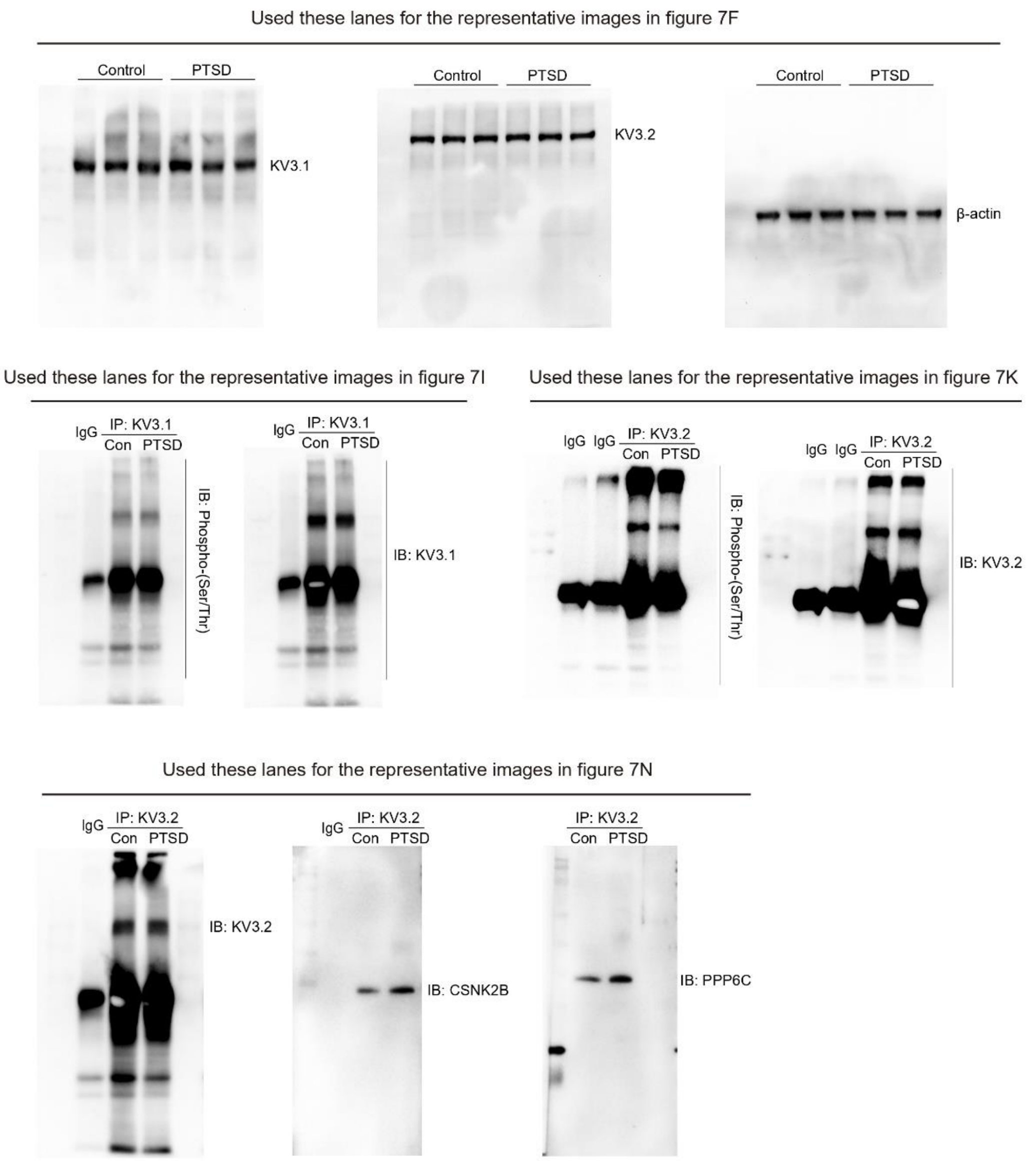

Figure S15. Full gel blot for Figure 7.

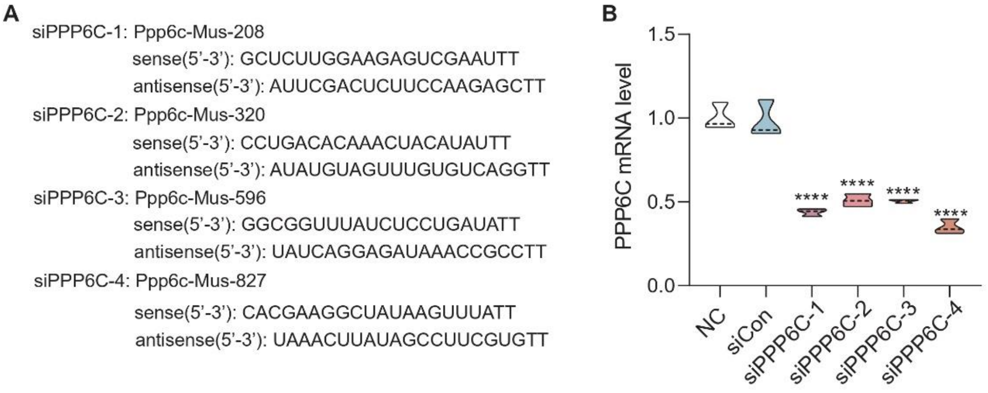

Figure S16. PPP6C siRNA screening. (**A**). The siRNA sequences. (**B**). Quantification of mRNA levels of PPP6C after administration with siPPP6C *in vitro.* n = 3 per group, error bar: mean with SEM, ****P < 0.0001 by one-way ANOVA with Tukey’s post hoc test. Statistical details are in Table S1.

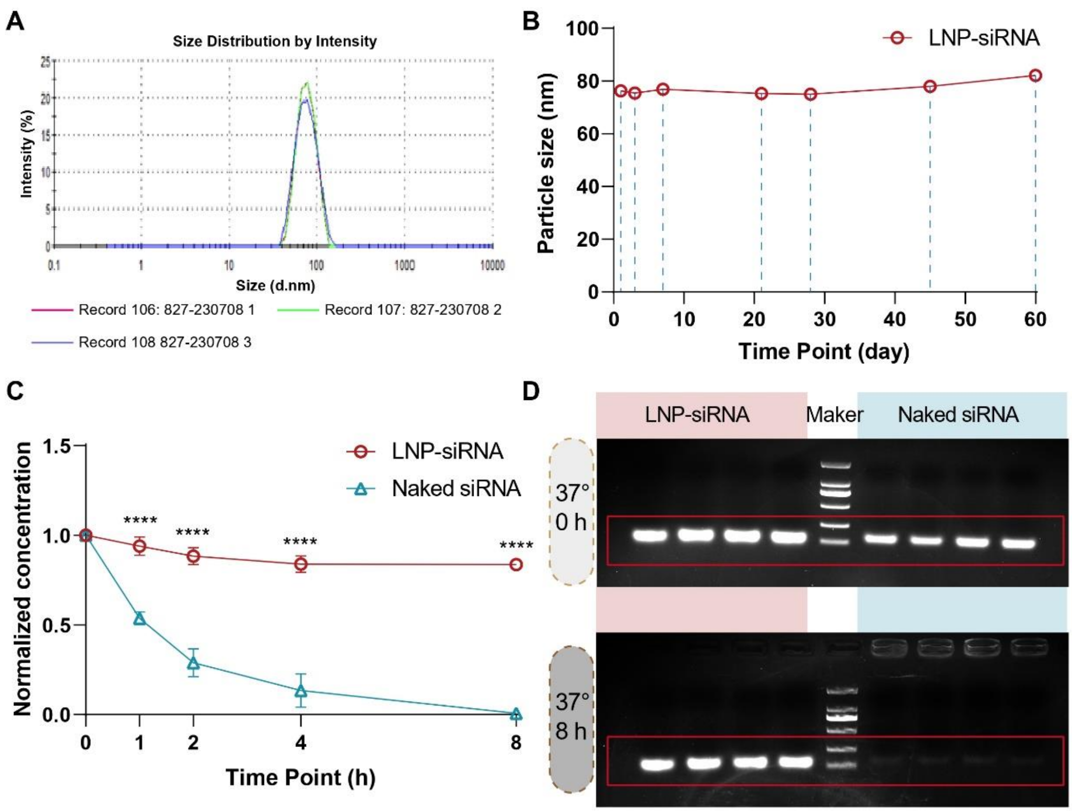

Figure S17. Comparison of the stability of LNP-siRNA and naked siRNA. (**A**). Size distribution by intensity. (**B**). The storage stability of LNP-siRNA. (**C**). *In vitro* degradation time of LNP-siRNA and naked siRNA. (n=3 per group, error bar: mean with SEM, ****$P < 0.0001$ by two-way ANOVA with Tukey's post hoc test). (**D**). Agarose gel electrophoresis results of LNP-siRNA and naked siRNA after 37° placement at 0 and 8 hours (n=4 per group). Statistical details are shown in Table S1.

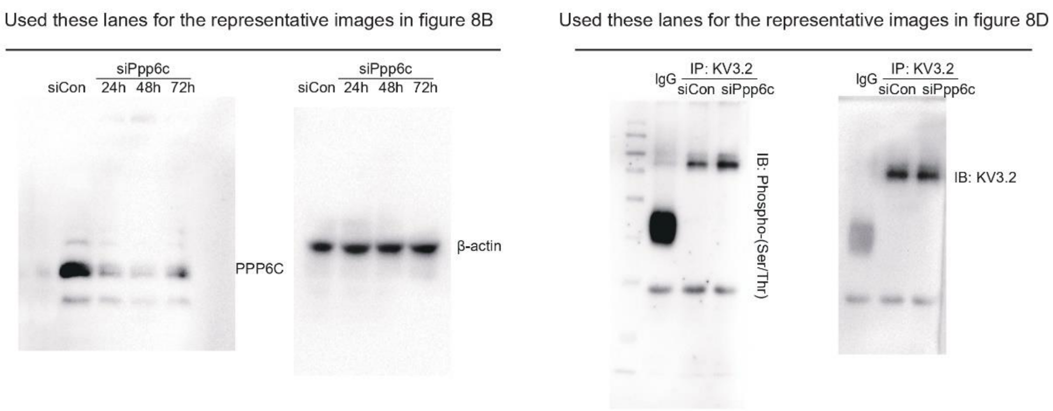

Figure S18. Full gel blot for Figure 8.

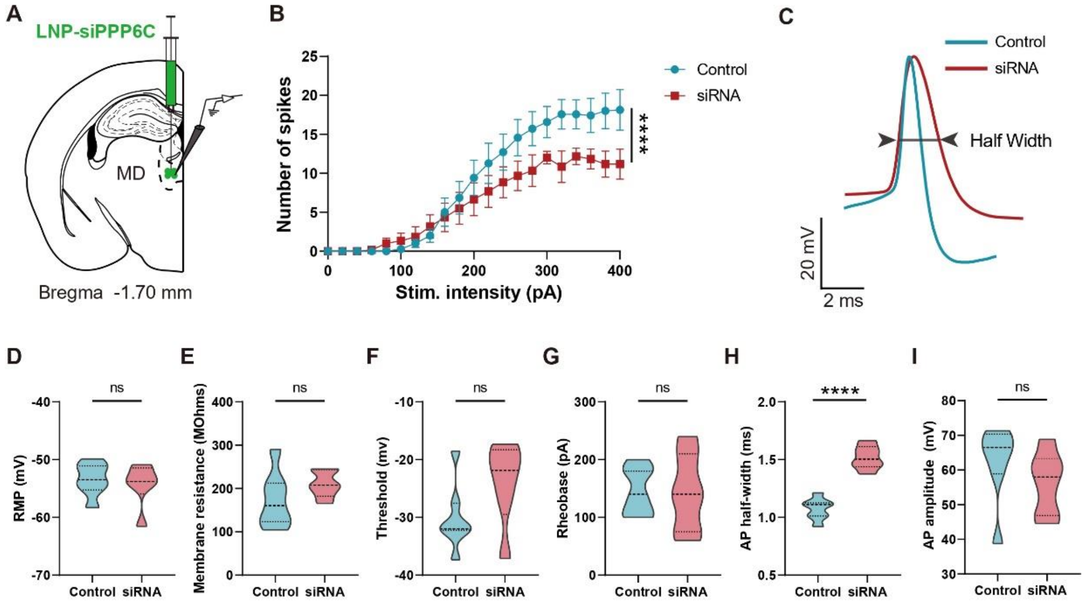

Figure S19. Action potential and active and passive properties of MD neurons after LNP administration. (**A**). Schematic of whole-cell patch in MD (**B**). Average number of spikes of neurons in MD between LNP-siRNA and LNP-empty groups in WT mice under current clamp (**C**). A representative image showing the half-width of MD neurons in LNP-siRNA and LNP-empty groups. (**D-I**). Quantification of active and passive properties of neurons in MD. n = 6–7 neurons from three mice per group, error bar: mean with SEM, ****$P < 0.0001$ by two-way ANOVA with Tukey's post hoc test, $ns_D = 0.6465$, $ns_E = 0.2442$, $ns_F = 0.1184$, $ns_G = 0.9398$, ****$P_H < 0.0001$ by two-tailed unpaired Student's t-test, $ns_I = 0.2343$ by Mann-Whitney U test. Statistical details are in Table S1.

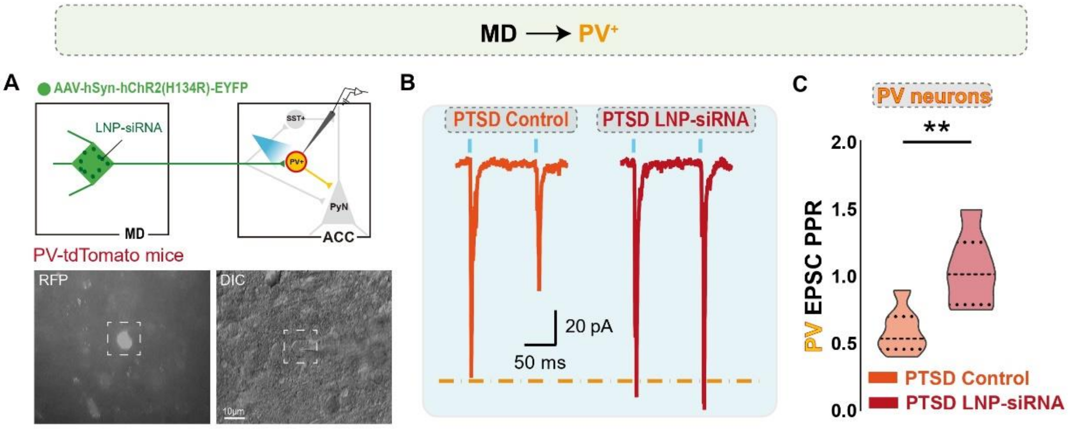

Figure S20. LNP-siRNA restored synaptic inputs from MD to ACC PV$^+$ neurons. (**A**). Schematics of the paired-pulse ratio procedure. Representative paired-pulse traces (**B**) and quantitative summary (**C**) of PV$^+$ neurons showing increased PPR in PTSD LNP-siRNA group (n = 7 neurons from three mice per group, error bar: mean with SEM, ***P* = 0.0022 by two-tailed unpaired Student's t-test). Statistical details are in Table S1.

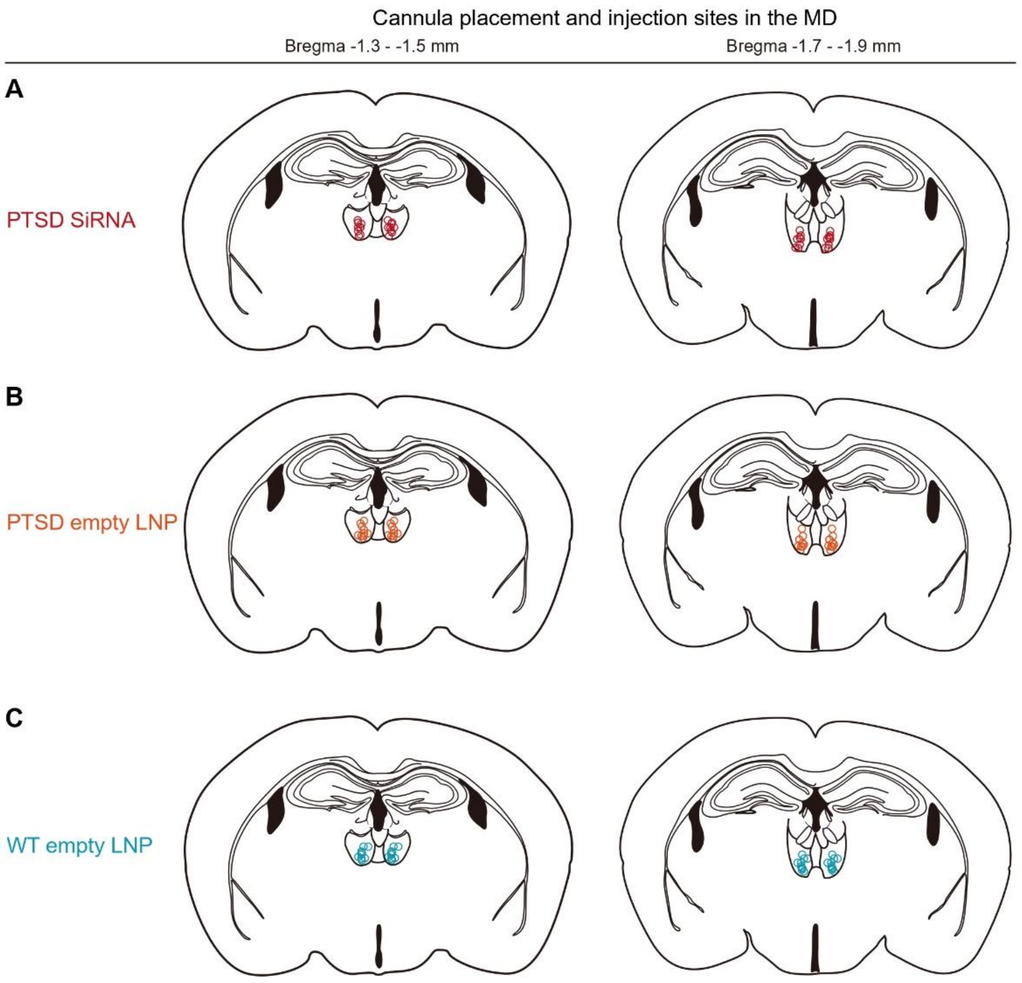

Figure S21. Cannula placement in MD. (**A-C**). Overlays of cannula tips in bilateral MD in pharmacological experiments related to **Fig. 8**. n = 10 mice per group.

Supplemental Table 1. Detailed statistical information.

Supplemental Table 2. Machine learning data for Figure 2.

Supplemental Table 3. Biophysical characteristics of lipid nanoparticles. Main characteristics of lipid nanoparticles (LNPs) used in this study, which are comprised of ionizable lipid (8-[(2-hydroxyethyl) [6-oxo-6-(undecyloxy) hexyl] amino]-octanoic acid, 1-octylnonyl ester (SM-102)), cholesterol, DSPC (1,2 distearoyl-sn-glycero-3-phosphocholine), α-[2-(ditetradecylamino)-2-oxoethyl]-ω-methoxy-poly(oxy-1,2-ethanediyl) (ALC-0159) and PPP6C siRNA. Data are shown as mean ± SEM for LNP-PPP6C siRNA (n= 3 independent measurements) and LNP-Control siRNA (n= 3 independent measurements). PDI: polydispersity index; EE (%): efficiency of encapsulation of the siRNA into LNPs. Size is given as the Z-average diameter.
