# Supplementary material for: Restoring the Function of Thalamocortical Circuit Through Correcting Thalamic Kv3.2 Channelopathy Normalizes Fear Extinction Impairments in a PTSD Mouse Model
Source: Adv Sci (Weinh). 2023 Dec 16;11(9):2305939. doi: 10.1002/advs.202305939 (PMC10916658; doi:10.1002/advs.202305939)
Supplement: Supplementary file 4 — Supplemental Table 3 [file ADVS-11-2305939-s002.pdf]

ADVANCED SCIENCE
Open Access

Supporting Information

for *Adv. Sci.*, DOI 10.1002/advs.202305939

Restoring the Function of Thalamocortical Circuit Through Correcting Thalamic Kv3.2 Channelopathy Normalizes Fear Extinction Impairments in a PTSD Mouse Model

Haoxiang Xiao, Kaiwen Xi, Kaifang Wang, Yongsheng Zhou, Baowen Dong, Jinyi Xie, Yuqiao Xie, Haifeng Zhang, Guaiguai Ma, Wenting Wang, Dayun Feng, Baolin Guo* and Shengxi Wu**

Lipid nanoparticles	Size (nm)	PDI	siRNA concentration (mg/mL)	EE (%)
**LNP-PPP6C siRNA**	73.18 ± 3	0.051 ± 0.01	0.91 ± 0.002	94 ± 0.2
**LNP-Control siRNA**	75 ± 4	0.077 ± 0.02	0.33 ± 0.003	96 ± 0.3

Supplemental Table 3. Biophysical characteristics of lipid nanoparticles. Main characteristics of lipid nanoparticles (LNPs) used in this study, which are comprised of ionizable lipid (8-[(2-hydroxyethyl) [6-oxo-6-(undecyloxy) hexyl] amino]-octanoic acid, 1-octylnonyl ester (SM-102)), cholesterol, DSPC (1,2 distearoyl-sn-glycero-3-phosphocholine), α-[2-(ditetradecylamino)-2-oxoethyl]-ω-methoxy-poly(oxy-1,2-ethanediyl) (ALC-0159) and PPP6C siRNA. Data are shown as mean ± SEM for LNP-PPP6C siRNA (n= 3 independent measurements) and LNP-Control siRNA (n= 3 independent measurements). PDI: polydispersity index; EE (%): efficiency of encapsulation of the siRNA into LNPs. Size is given as the Z-average diameter.
